# Epicardial and endothelial cell activation concurs with extracellular matrix remodeling in atrial fibrillation

**DOI:** 10.1002/ctm2.558

**Published:** 2021-11-04

**Authors:** Nicoline W. E. van den Berg, Makiri Kawasaki, Benedetta Fabrizi, Fransisca A. Nariswari, Arianne C. Verduijn, Jolien Neefs, Robin Wesselink, Rushd F. M. Al‐Shama, Allard C. van der Wal, Onno J. de Boer, Jan Aten, Antoine H. G. Driessen, Aldo Jongejan, Joris R. de Groot

**Affiliations:** ^1^ Department of Clinical and Experimental Cardiology, Amsterdam Cardiovascular Sciences Amsterdam UMC, University of Amsterdam, Heart Center Amsterdam The Netherlands; ^2^ Department of Clinical Pathology Amsterdam UMC, University of Amsterdam Amsterdam The Netherlands; ^3^ Department of Epidemiology & Data Science Amsterdam UMC, University of Amsterdam Amsterdam The Netherlands

**Keywords:** angiogenesis, arrhythmias, atrial fibrillation, atrial remodeling, epithelial‐to‐mesenchymal transition, extracellular matrix, transcriptome

## Abstract

**Background:**

Improved understanding of the interconnectedness of structural remodeling processes in atrial fibrillation (AF) in patients could identify targets for future therapies.

**Methods:**

We present transcriptome sequencing of atrial tissues of patients without AF, with paroxysmal AF, and persistent AF (total *n* = 64). RNA expression levels were validated in the same and an independent cohort with qPCR. Biological processes were assessed with histological and immunohistochemical analyses.

**Results:**

In AF patients, epicardial cell gene expression decreased, contrasting with an upregulation of epithelial‐to‐mesenchymal transition (EMT) and mesenchymal cell gene expression. Immunohistochemistry demonstrated thickening of the epicardium and an increased proportion of (myo)fibroblast‐like cells in the myocardium, supporting enhanced EMT in AF. We furthermore report an upregulation of endothelial cell proliferation, angiogenesis, and endothelial signaling. EMT and endothelial cell proliferation concurred with increased interstitial (myo)fibroblast‐like cells and extracellular matrix gene expression including enhanced tenascin‐C, thrombospondins, biglycan, and versican. Morphological analyses discovered increased and redistributed glycosaminoglycans and collagens in the atria of AF patients. Signaling pathways, including cell–matrix interactions, PI3K‐AKT, and Notch signaling that could regulate mesenchymal cell activation, were upregulated.

**Conclusion:**

Our results suggest that EMT and endothelial cell proliferation work in concert and characterize the (myo)fibroblast recruitment and ECM remodeling of AF. These processes could guide future research toward the discovery of targets for AF therapy.

## Graphical abstract

Various signaling pathways are upregulated in atrial fibrillation, which can activate epithelial‐to‐mesenchymal transition and endothelial cell proliferation and differentiation. These processes are associated with increased interstitial fibroblasts and increased and altered extracellular matrix deposits including glycoproteins and glycosaminoglycans. EC, endothelial cell; ECM, extracellular matrix; EMT, epithelial‐to‐mesenchymal transition; FB, fibroblast; MSC, mesenchymal cells; PVC, perivascular cell.

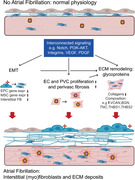

## 1 BACKGROUND

Atrial fibrillation (AF) is the most common cardiac arrhythmia, and the fast growing prevalence and burden on health care systems call for the identification of new targets for therapy.^1^, ^2^ Current medical therapies aim to prevent AF recurrence or AF‐related complications by modifying electrophysiological targets or inhibiting coagulation, but no clinical therapies are available that effectively target structural remodeling of the AF substrate.^3^

Atrial structural remodeling precedes the first onset of AF and is associated with AF progression.^4^, ^5^, ^6^ However, the key processes underlying the AF substrate remain incompletely understood. Structural remodeling generally refers to atrial fibrosis: a pathological accumulation of extracellular matrix (ECM) proteins, among which type I collagen and type III collagen are most abundant.^7^ Structural remodeling encompasses numerous ECM proteins and involves a complex concert of (patho)physiological processes. The ECM provides structural support to the cardiomyocytes and is involved in signaling that can modulate cell proliferation, migration, and adhesion.^8^ During fibrosis formation, ECM is mainly deposited by proliferating and activated interstitial (myo)fibroblasts.^9^, ^10^ The origin of cardiac fibroblasts in structural remodeling is under debate and may vary depending on the underlying pathophysiology.^11^, ^12^, ^13^ In AF, the epicardium, containing mesenchymal progenitor cells has been identified as a potential source of fibroblasts following cardiac injury through differentiation and migration in a process referred to as epithelial‐to‐mesenchymal transition (EMT).^14^, ^15^ Mesenchymal progenitor cell differentiation leads to a dynamic display of different mesenchymal cell phenotypes, and can give rise to adipocytes, fibroblasts, (myo)fibroblasts, and perivascular cells.^16^ Differentiated fibroblast‐like cells constantly form and degrade ECM, underpinning that ECM is a highly dynamic structure.

Our current knowledge of atrial structural remodeling is fragmented as most of the published literature focuses on single biological pathways. With respect to studies in patients and human materials, these are commonly faced with practical restrictions. First, materials derived from cardiothoracic surgery for other conditions than AF are commonly used. As a consequence, there are relatively many reports on, for example, mitral stenosis‐related AF, although this condition is clinically uncommon in the developed world.^17^ Second, human tissues are typically available at only one point in time, which complicates the study of continuous substrate remodeling, opposed to remodeling that may be specific to the different types of AF.

In the current study, we profiled left atrial tissue samples of patients undergoing thoracoscopic surgery for AF as their primary diagnosis. As controls, we used left atrial tissues from patients without AF undergoing unrelated cardiothoracic surgery. By profiling a large population of clinically well‐defined patients including both paroxysmal and persistent AF patients, we aimed to distinguish processes that play a role throughout the course of the disease from those that are disease stage specific.

We used transcriptome sequencing to examine the changes in non‐cardiomyocyte mesenchymal gene expression and changes in ECM components beyond collagens. We sought to describe how multiple structural remodeling processes coexist and interconnect in patients with AF at different stages of the disease.

## 2 METHODS

### 2.1 Patient recruitment and ethical approval

We included 22 patients without a history of AF (non‐AF), 22 patients with paroxysmal AF (par‐AF), 18 patients with persistent AF (pers‐AF), and two longstanding persistent AF (lspers‐AF) patients. Paroxysmal, persistent, and longstanding persistent AF were defined according to current guidelines,^3^ though for the current analysis patients with pers‐AF and lspers‐AF were pooled together in pers‐AF.

**HIGHLIGHTS**

- Atrial structural remodeling in atrial fibrillation is accompanied by a widespread activation of non‐cardiomyocyte mesenchymal cells found in the epicardium and perivascular niche.
- Atrial extracellular matrix remodeling not only consists of collagen synthesis, but involves glycoproteins, proteoglycans, and increased extracellular matrix turnover.
- Atrial fibrillation is accompanied by hypoxic signaling, angiogenesis, and increased microvessel density.
- Numerous signaling pathways involved in epithelial‐to‐mesenchymal transition and structural remodeling are upregulated in human atrial fibrillation, which suggests a complex interplay of parallel pathways.

Control subjects without a history of AF participated in the previously described PREDICT‐AF study (NCT03130985 April 27, 2017).^6^, ^18^ In the PREDICT‐AF study, 150 patients without a history of AF, who underwent cardiothoracic surgery for coronary artery bypass grafting (CABG) and/or valve surgery between 2015 and 2018, were prospectively followed to determine the development of incident AF. As part of the study, the left atrial appendage (LAA) was removed for molecular and histopathological analyses. Eligible participants had a CHA_2_DS_2_‐VASc score of ≥2 and were aged 18–80 years.

Patients with AF participated in the AFACT trial (NCT01091389) and MARK AF registry.^19^ Both studies applied the same inclusion and exclusion criteria to determine the eligibility of patients with par‐AF or pers‐AF undergoing thoracoscopic AF ablation. The LAA was excised as part of routine care in all patients for thrombosis prophylaxis. All excised LAAs were stored in the ADAPT biobank (NCT04776642) for the discovery of biomarkers.

All subjects were clinically characterized, underwent preoperative screening and echocardiography prior to surgery. All patients provided informed consent. Tissue removal and tissue storage were conducted in accordance with the protocol. The protocol was approved by both the Institutional Review Board and the Biobank Review Board of the Amsterdam University Medical Center location AMC and was in accordance with the Declaration of Helsinki.

### 2.2 Tissue processing and transcriptome sequencing

Upon excision, LAAs were directly washed in ice‐cold modified Tyrode's solution before one half was fixated in formaldehyde and the other half was snap‐frozen in liquid nitrogen in the operating theater and stored at −80°C. We extracted RNA from 50–100 mg of snap frozen whole tissues using Trizol (Invitrogen, Cat. No. 15596018). The quality of the RNA samples used for whole transcriptome sequencing was confirmed by Bioanalyzer (RIN values 8.4 ± 0.9; Agilent 2100, CA, USA). Samples were rRNA depleted and used for paired end sequencing (100 bp) on the Illumina NovaSeq 6000 (Illumina, CA, USA) (details in Supporting Materials). For further analyses, we selected protein coding genes only.

### 2.3 Data alignment and computation analysis

Reads were mapped toward the human reference genome (GRCh38) using HiSAT2 v2.1.0 and counted with HTSeq v0.11. The differential expression analysis compared three study groups: control, par‐AF, and pers‐AF, and was corrected for age and gender (R package *Voom*). Genes with a false discovery rate (FDR) adjusted *p *< .05 (Benjamini–Hochberg) were considered to be differentially expressed genes (DEG) unless otherwise specified. Displayed fold‐changes (FCs) are log2 transformed as are reported counts per million (CPM). We focused on the processes that were continuously increasing or decreasing from non‐AF to par‐AF to pers‐AF. If not otherwise indicated, the FCs and FDRs that were reported resulted from comparing pers‐AF to non‐AF.

### 2.4 Gene set enrichment

Competitive gene set enrichment analysis (GSEA) was performed for all three comparisons using a ranked gene list: sign(log2FC)*−log10(FDR). Details regarding the strategy for GSEA have been described previously.^20^ GSEA was performed searching the MSigDB gene set database (v September 2019) for biological processes, KEGG pathways, and transcription factor (TF) targets. Significance of enrichment scores was determined using a phenotype permutation (1000×) and was set at a FDR *q*‐value of .05. Visualization was performed using the cytoscape plugin *Enrichmentmap*,^21^ and KEGG pathways were visualized with the R package *pathview*. GSEA produces *p*‐values of absolute zero for extremely low values. For the purpose of comparison and visualization, these were imputed with the value .00001.^20^

### 2.5 Quantitative polymerase chain reaction (qPCR) validation of DEGs

Gene expression results were validated by qPCR in the study cohort and an independent cohort including non‐AF, par‐AF, and pers‐AF patients (total *n* = 30) with similar clinical characteristics. Baseline characteristics of the independent cohort are described in Table S1.

cDNA was synthesized from the same pool of total RNA as used for RNA sequencing. For biological validation, RNA was extracted from tissue samples of the independent cohort. cDNA was synthesized from 500 ng of total RNA with SuperScript II reverse transcriptase (Invitrogen, Cat. No. 18064022). Real‐time PCR quantification was performed on the LightCycler 480 (Roche) with the SYBR Green PCR Kit (Roche, Cat. No. 04707516001). Starting concentrations of each gene were calculated using LinRegPCR.^22^ Values were normalized against the geometric mean of *GUSB, HPRT1*, and *PGK1*, which were selected with the NormFinder algorithm after testing five candidates (*GUSB, HPRT1, PGK1, RPL32, POLR2A*) that had been identified from literature and had been assessed for their expression in the sequencing data.^23^, ^24^, ^25^ Used primers are given in Table S2.

### 2.6 Histological and immunohistological analyses

In 61 out of 64 patients, left atrial tissue was available for histological analyses. Transmural paraffin sections were prepared at a thickness of 5 μm.

Immunohistochemistry of CD31 (Dako M0823 clone JC70A, 1:250), vimentin (Dako M0725 clone V9IgG1, 1:1000), and αSMA (Dako M0851 clone 1A4, 1:800) was performed with the Ventana Benchmark Ultra autostainer. Immunohistochemistry of fibroblast specific protein (FSP1, S100A4 Dako A5114, 1:2500), SNAIL (Abcam, ab17732, 1:250), TWIST (Santa Cruz Biotechnology, sc‐15393, 1:100), NFAT2 (Abcam, ab2796, 1:50), WT1 (Abcam, ab89901, 1:300), and CD44 (Abcam, ab24504, 1:2500) was performed manually and visualized using DAB (Immunologic BrightDAB BS04). Immunohistochemistry of type I collagen (Abcam EPR7785 ab138492, 1:1000) was visualized with NovaRed (Vector Laboratories Inc. #SK‐4800) and subsequently digitized, stripped (β‐mercaptoehtanol in Tris‐HCl), and re‐stained with type III collagen (Biogenex HWD1.1, 1:200). In a similar manner, sections were stained successively with CD31, αSMA, and vimentin, and with αSMA, FSP1, and vimentin.

Sections were stained with alcian blue (Alfa Aesar J60122 lotnr Q24F022) and nuclear fast red (Clin‐Tech 642420 lotnr C153‐22‐06) and with picrosirius red (Sirius red F3B, #34149‐2F, BDH, Brunschwig).

All sections were digitized at a 40× magnification (Philips IntelliSite Ultra Fast Scanner, 0.25 μm/pixel). Random fields from qualitatively good stainings were selected for quantitative analyses. The endocardial layer, epicardial layer, and major vessels were manually excluded prior to analyses in imageJ (Color Deconvolution).

Sections stained on the autostainer (CD31, Vimentin, αSMA) were quantified by normalizing the positively stained area for the interstitial cardiomyocyte count (nuclei). FSP1 was quantified by normalizing the positively stained area against the total area selected for analysis.

Alcian blue stainings were quantified by dividing the glycosaminoglycan area (blue) by the total area selected for analysis. The interstitial distribution of proteoglycans was categorized by two independent reviewers as “none,” “moderate,” “extensive” endocardial localization.

Picrosirius red staining was quantified by dividing the area of collagen (red) by the area of cardiomyocytes (yellow). A two‐step approach was applied to quantify perivascular collagens, by quantifying sections prior to and after the manual removal of larger vessels.

### 2.7 Statistical analysis

Histological and qPCR data were compared with ANOVA for normally distributed data, and Kruskal–Wallis test for non‐normally distributed data. We compared non‐AF, par‐AF, and pers‐AF patient groups. All performed tests were two‐sided and a *p*‐value <.05 was considered statistically significant (R version 3.2.3).

## 3 RESULTS

### 3.1 Differential gene expression increases between no, paroxysmal, and persistent AF

The atrial tissues from non‐AF (*n* = 22), paroxysmal AF (*n* = 22), and persistent AF (*n* = 20) patients were used for transcriptome analysis. The baseline characteristics of the patients are displayed in Table 1.

**TABLE 1:** Patient characteristics

	**All patients *n* = 64**	**Non‐AF *n* = 22**	**Par‐AF *n* = 22**	**Pers‐AF *n* = 20**
Sex, female, *n* (%)	15 (23.4)	6 (27.3)	5 (22.7)	4 (20.0)
Age, years (±SD)	63.0 ± 9.3	66.8 ± 8.4	60.0 ± 8.3	62.2 ± 10.3
BMI, kg/m^2^ (±SD)	27.1 ± 3.4	28.1 ± 3.3	26.1 ± 3.9	26.9 ± 2.8
Myocardial infarction, *n* (%)	8 (12.5)	5 (22.7)	1 (4.6)	2 (10.0)
PCI, *n* (%)	5 (7.8)	3 (13.6)	1 (4.6)	1 (5.0)
CHA_2_DS_2_‐VASc [IQs]	2 [1–3]	3 [2.25–4]	1 [0.25–1.75]	1 [0.25–1.75]
CHA_2_DS_2_‐VASc ≥4	9 (14)	7 (32)	1 (4.5)	1 (5.0)
Vascular disease, *n* (%)	22 (34.4)	17 (77.3)	1 (4.6)	4 (20)
Hypertension, *n* (%)	35 (54.7)	17 (77.3)	8 (36.4)	10 (50.0)
Diabetes mellitus, *n* (%)	6 (9.4)	5 (22.7)	0	1 (5.0)
Congestive heart failure, *n* (%)	0	0	0	0
Stroke/TIA/embolus, *n* (%)	10 (15.6)	6 (27.3)	2 (9.1)	2 (10.0)
Echocardiography				
Max LAVI, ml/m^2^ (±SD)	36.3 ± 12	31.4 ± 9	36.1 ± 11	42.4 ± 14
LVEF, % (±SD)	53.6 ± 11	48.4 ± 11	58.6 ± 10	54.0 ± 10
Medication				
Antiplatelet, *n* (%)	20 (31.3)	19 (86.4)	1 (4.6)	0
Anticoagulation, *n* (%)	41 (64.1)	0	22 (100)	20 (100)
ACE inhibitors, *n* (%)	21 (32.8)	10 (45.5)	6 (27.3)	5 (25)
Angiotensin receptor blockers, *n* (%)	15 (23.4)	5 (22.7)	4 (18.2)	6 (30)
Class IA AAD	5 (7.8)	0	4 (18.2)	1 (5.0)
Class IC AAD	17 (26.6)	0	9 (40.9)	8 (40.0)
Class II AAD	31 (48.8)	12 (54.5)	9 (40.9)	10 (50.0)
Class III AAD	13 (20.3)	1 (4.6)	8 (36.4)	4 (20.0)
Class IV AAD	5 (7.8)	0	2 (9.1)	3 (15.0)
Digoxine, *n* (%)	8 (12.5)	0	3 (13.6)	5 (25.0)

Abbreviations: AAD, antiarrhythmic drugs; ACE, angiotensin converting enzyme; AF, atrial fibrillation; BMI, body mass index; IQ, interquartiles; LAVI, left atrial volume index; LVEF, left ventricular ejection fraction; PCI, percutaneous coronary intervention; SD, standard deviation; TIA, transient ischemic attack.

**FIGURE 1:**
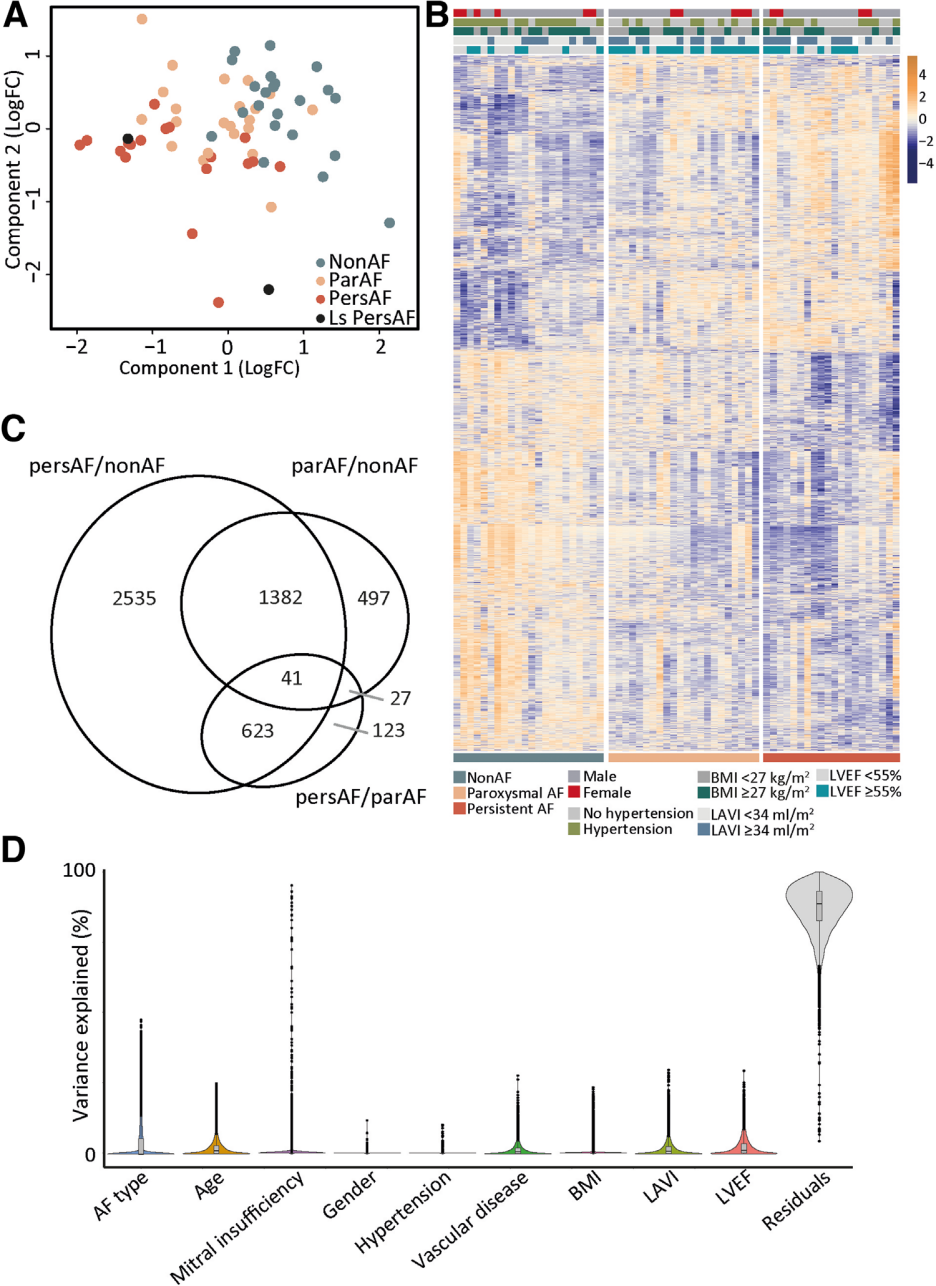
Human atrial gene signature identifies common and disease stage‐specific expression. (A) Dimensionality reduction showed a separation of non‐AF, par‐AF, and pers‐AF atrial samples. (B) Semi‐supervised heatmap of all DEG showing an ordinal increase in gene expression levels and lack of clustering by clinical characteristics. (C) Number and overlap of DEG found for each comparison. (D) Variation partitioning demonstrating that there is not a single clinical characteristic that has a major impact on gene expression. BMI, body mass index; LAVI, left atrial volume index; LVEF, left ventricular ejection fraction; sign, significant

We found 17,324 unique protein coding genes expressed in the left atrium. Among these, 5228 were differentially expressed between the three comparisons; par‐AF versus non‐AF, pers‐AF versus par‐AF, and pers‐AF versus non‐AF. Dimensionality reduction showed a tended separation of the study groups based on their gene signatures (Figure 1A). A heatmap of the DEG showed the gradual increase or decrease of DEG from non‐AF to par‐AF to pers‐AF (Figure 1B). Two patients with longstanding pers‐AF had been included, but did not clearly separate from pers‐AF patients after dimensionality reduction (Figure 1A) or nonsupervised hierarchical clustering (Figure S1) and were therefore pooled with pers‐AF patients. More than 85% of DEGs were embedded in the comparison pers‐AF versus non‐AF, supporting the notion that AF gene signatures follow an ordinal scale from non‐AF to par‐AF to pers‐AF (Figure 1C). A complete list of DEGs is displayed in the Supporting Data File.

Patients did not cluster based on clinical characteristics or comorbidities with unsupervised hierarchical clustering per study group (Figure 1B and Figure S1), and none of the clinical characteristics significantly affected gene expression signatures (Figure 1D).

### 3.2 Gene signatures point to epicardial cell loss and mesenchymal cell increase in left atria of patients with AF

**FIGURE 2:**
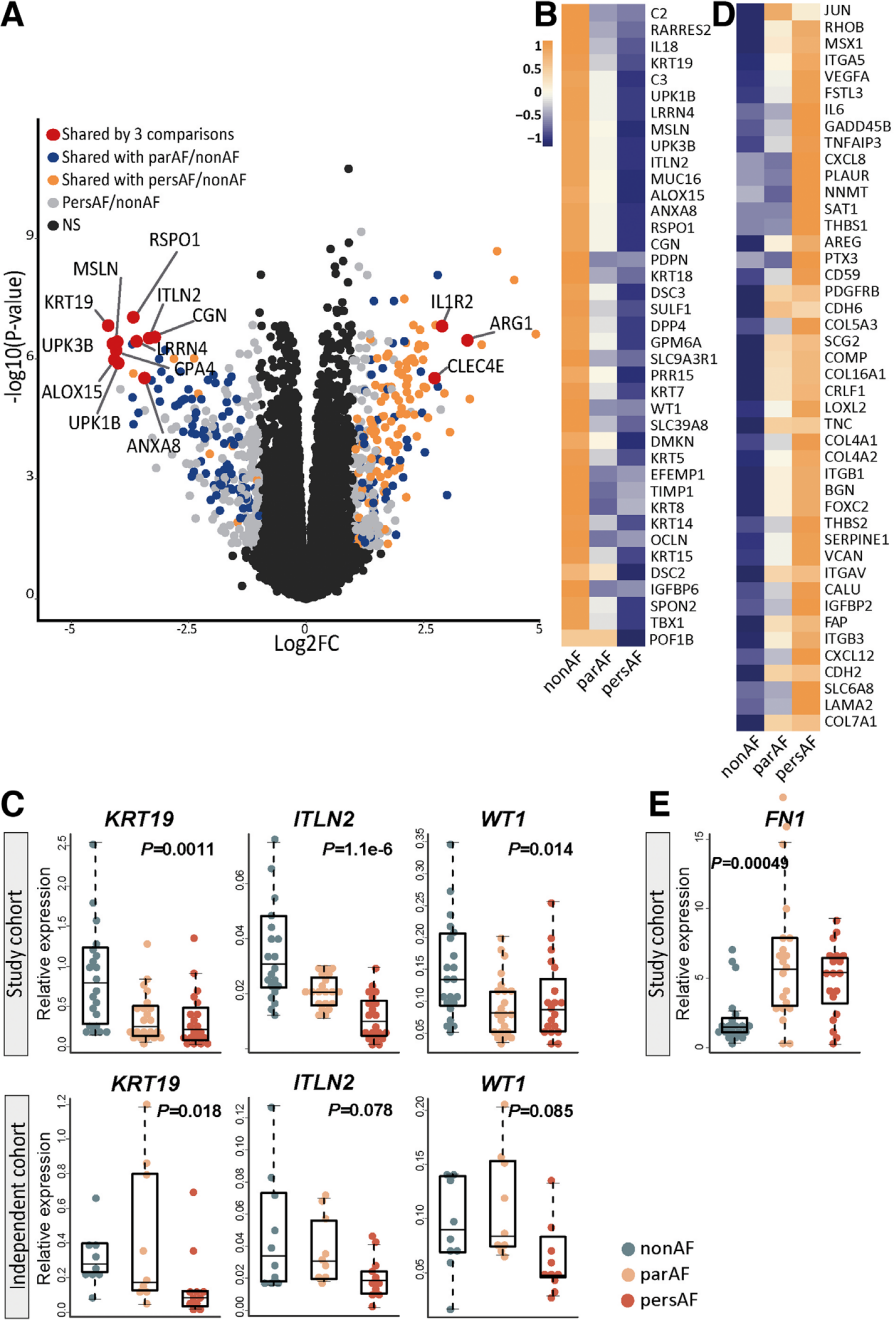
Decreased epicardial gene expression reveals epithelial‐to‐mesenchymal transition. (A) Volcano plot of the comparison pers‐AF versus non‐AF identified genes shared by all three comparisons (i.e., par‐AF vs. non‐AF, pers‐AF vs. par‐AF, and pers‐AF vs. non‐AF) that were mostly downregulated epicardial enriched genes. (B) Broad downregulation of epicardial marker genes was found (FDR <0.05). (C) qPCR‐validated epicardial cell gene downregulation in the study cohort and an independent cohort. (D) DEG of the upregulated biological process “Hallmark epithelial‐to‐mesenchymal transition” (MSigDB). (E) Mesenchymal marker fibronectin was highly upregulated. Boxplots depict range and interquartile range

The majority of most significantly DEGs shared by all three comparisons were downregulated and showed enrichment in epicardial adipose tissues (EAT) or, more specifically, the epicardial cell layer (*MSLN*, *LRRN4*, *UPK3B*, *UPK1B*, *KRT19, RSPO1, ALOX15, ITLN2, ANXA8, CGN*; all showed at least a log2 FC <−3 and FDR <5.8^E−6^) (Figure 2A).^26^, ^27^, ^28^ The profound downregulation of these epicardial cell (EPC) genes in AF uncovered an extensive downregulation of various EPC markers and of epithelial cell–cell junction genes (Figure 2B), including cytokeratins and cadherins (*KRT14, KRT18, CDH3*).^15^ E‐cadherin (*CDH1*) was moderately decreased (FC = −0.77, FDR = 0.065). Downregulation of EPC genes was corroborated by qPCR in the same patients and in an independent study cohort (Figure 2C).

A decrease in epithelial cell gene expression and a dissolution of epithelial cell–cell junctions characterize the initial processes of EMT.^15^ Indeed, among the upregulated biological processes identified by GSEA were *cardiac EMT* and *Hallmark EMT* (Figure 2D). Within these biological processes, genes frequently expressed by non‐cardiomyocyte mesenchymal cells, such as fibroblasts and myofibroblasts (hereafter MSC), were increased (e.g., *CDH2, MMP9, ITGAV*; Figure 2D). Fibronectin (*FN1)*, expressed by MSC, was validated with qPCR to be highly increased in par‐AF and pers‐AF (*p *= .00049; Figure 2E).^15^

Among the established TFs of EMT were increased *SNAI1, ZEB1, FOXC2, FOXF1*, and *NFATC1* in AF patients, whereas expression levels of *WT1, TWIST2*, and *ALDH1A2* were decreased^15^, ^16^ (Figure S2).

### 3.3 Histological changes support EMT

We further examined the epicardial layer for morphological changes characteristic of EMT, such as a changes in cell shape resulting from a reorganization of the cytoskeleton.^15^ In non‐AF and AF patients, the left atrial epicardial layer was a single‐layered epicardium, lining EAT or subepicardial connective tissue of varying thickness. The subepicardial layer was highly enriched in type I collagen and type III collagen (Figure 3A).^29^ In AF patients, the vimentin‐positive epicardial cell monolayer was thickened and disorganized in clustered areas (Figure 3B). αSMA in the epicardial monolayer did not differ between non‐AF and AF patients (Figure 3C), but interstitial αSMA increased (details below).

FIGURE 3Morphological changes of the epicardium support epithelial‐to‐mesenchymal transition. (A) Immunohistochemistry of type I and type III collagen and the merged image of the two stainings illustrate the relation between the epicardial cells, the fibrous subepicardium, and epicardial adipose tissue. The subepicardium is highly enriched with collagen 1 and collagen III fibers. Type I collagen is found directly lining the epicardial monolayer. (B) Vimentin‐stained sections identify the epicardial monolayer and (interstitial) myocardial vimentin+ cells. In AF, there is thickening of the epicardium and a loss of epicardial cell organization. (C) αSMA+ cells (arrows) were found in the epicardium of non‐AF and AF patients. (D) WT1+ cells were found in the epicardial monolayer and subepicardial fibrotic layer in both non‐AF and pers‐AF patients. Absolute number of WT+ appeared decreased in pers‐AF (compare a decrease in Vim+ cells in J). Ratio of cells residing in the subepicardium (arrows) compared to the epicardial monolayer increased in pers‐AF. (E) NFATC1 was found in the epicardium and subepicardium of AF patients. Note the clusters of subepicardial NFATC1+ cells in pers‐AF (arrow). Almost no signals were observed in non‐AF. (F) Number of SNAIL+ cells increased in AF patients along with thickening of the epicardial monolayer. Arrows indicate SNAIL+ nuclei localized to the apical layer, suggesting a loss of polarization. (G) TWIST was lower expressed and cytoplasmic (arrows) in AF patients. (H) Quantification of the area of the fibrous subepicardium identified a tended increase in pers‐AF. (I) EAT area did not differ between the study groups, although the spread in pers‐AF patients was remarkable. (J) A tended decrease in subepicardial vimentin+ area fraction was found in par‐AF and pers‐AF. (K and L) Interstitial myocardial vimentin+ area fraction increased in pers‐AF. (M and N) Sections stained with the fibroblast‐specific protein (FSP1) demonstrated an increased FSP1+ area fraction in AF. CM, cardiomyocyte; EAT, epicardial adipose tissue; EPC, epicardial cell genes; MSC, mesenchymal cells; Vim, vimentin. Boxplots depict range and interquartile range

**Figure:**
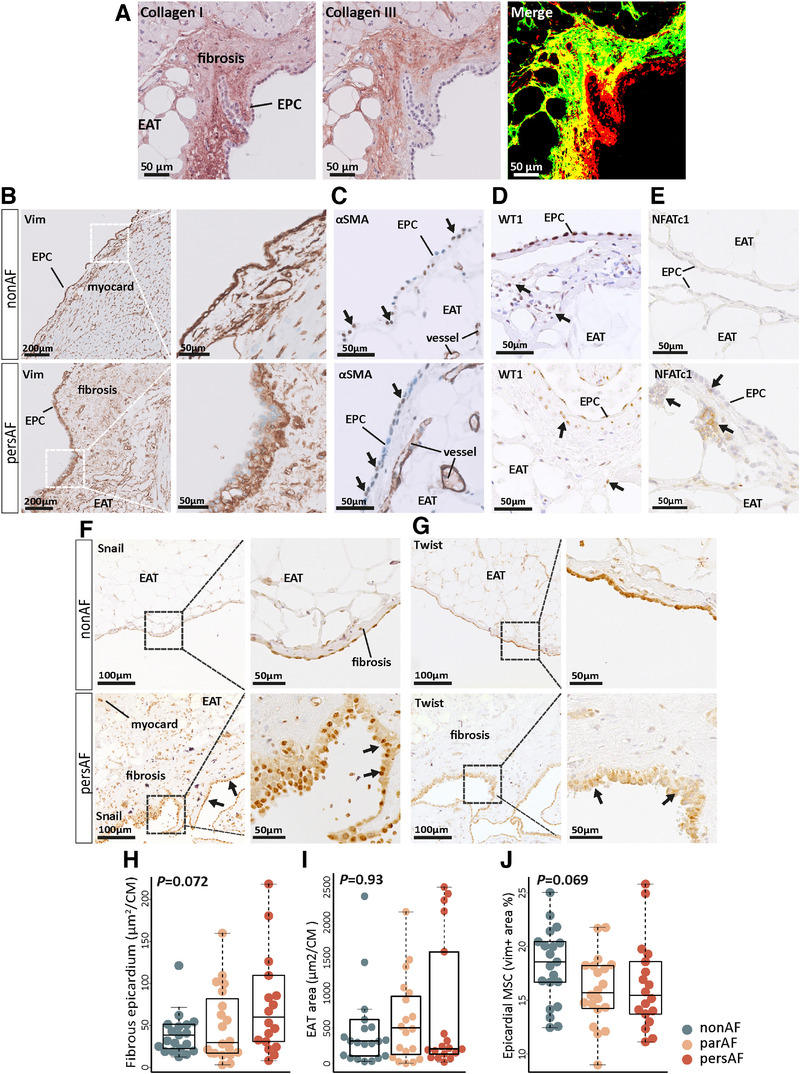

**Figure:**
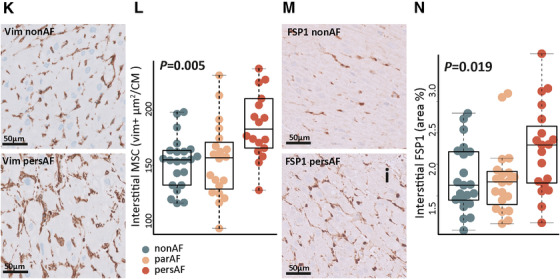

Gene expression of *WT1* was lower in AF patients, which was confirmed by immunohistochemistry (Figure 3D). In AF patients, a relatively larger number of WT1‐positive cells resided in the subepicardium compared to the epicardial monolayer. NFATC1 was seen in the epicardial monolayer and in clusters of subepicardial cells in AF patients. Almost no NFATC1 signals were observed in non‐AF (Figure 3E). The absolute number of epicardial cells expressing SNAIL increased in AF patients, along with thickening of the epicardial layer. Nuclear SNAIL localized toward the apical layer in AF patients, suggesting a loss of epithelial cell polarization (Figure 3F).^30^ Immunohistochemistry of TWIST showed a lower intensity and cytosolic localization in AF patients (Figure 3G). These observations were consistent with the transcriptome data and GSEA results (Supporting Data File).

There was a trend toward an increased fibrous subepicardial area in pers‐AF patients (Figure 3H), consistent with the activation of (sub)epicardial MSC and increased ECM deposits.^14^ No difference in EAT area was observed between AF and non‐AF patients (Figure 3I and Figure S3). However, the numerical spread in EAT area in pers‐AF patients was large and suggestive of two distinct groups within the pers‐AF patients, which was not driven by lspers‐AF.

In contrast to a tended decrease in vimentin‐positive area in the fibrous subepicardium (Figure 3I), the interstitial vimentin‐positive area significantly increased in AF patients compared to non‐AF patients (Figure 3J,K). Fibroblast‐like cells expressing fibroblast specific protein (FSP1) were increased in the interstitium of atrial sections of pers‐AF compared to non‐AF (Figure 3L,M). These observations are consistent with migration of epicardial‐derived cells to the interstitial myocardium.

### 3.4 Gene signatures point to increased angiogenesis

**FIGURE 4:**
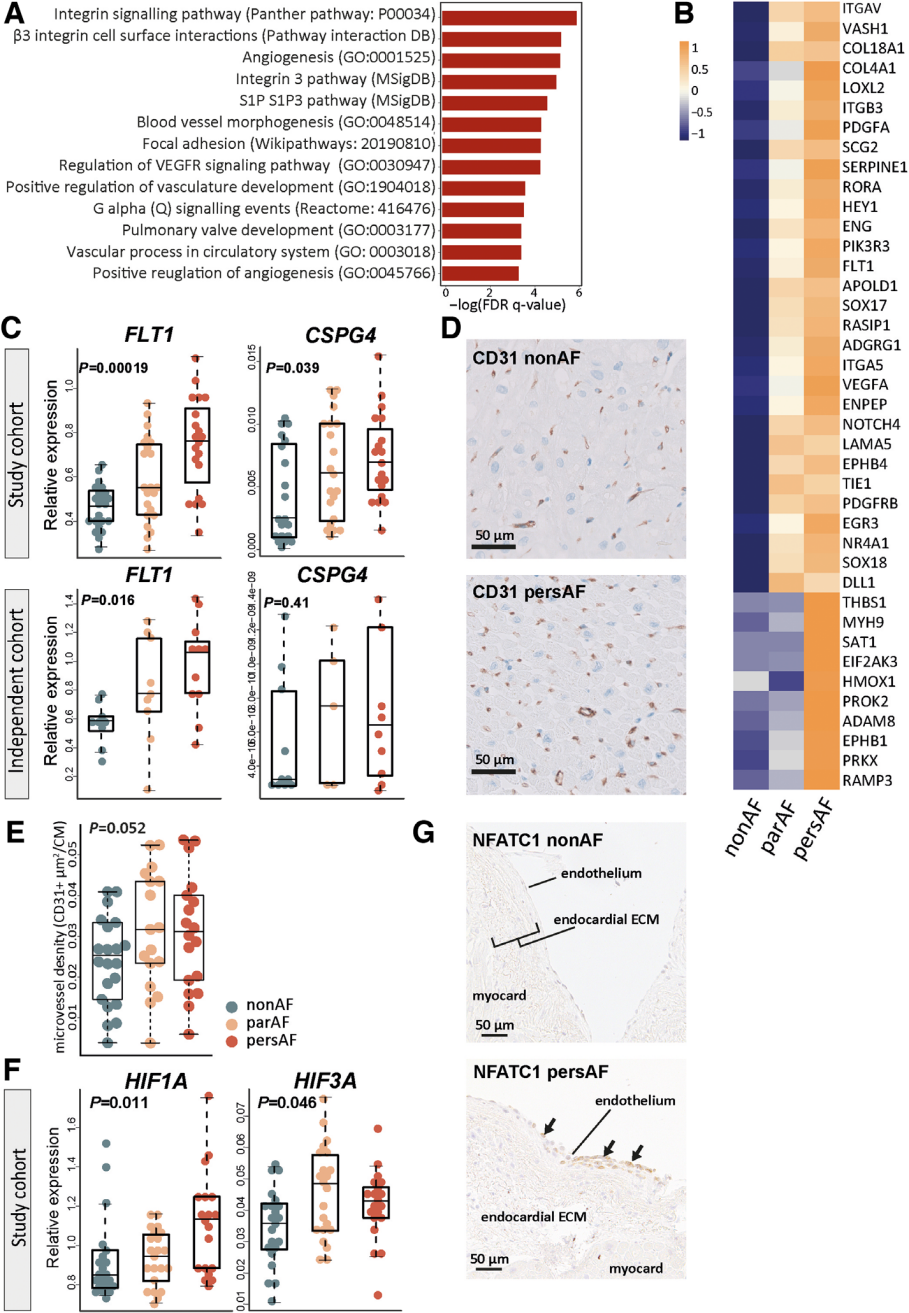
Angiogenesis is increased throughout the course of atrial fibrillation. (A) The upregulated biological processes shared by all three comparisons related to endothelial cell proliferation, endothelial signaling, and angiogenesis. Presented enrichment scores and FDR *q*‐values result from the comparison pers‐AF versus non‐AF. (B) Heatmap showing the leading edge genes of angiogenesis (GO:0001525). (C) qPCR validated the upregulation of *FLT1* and *CSPG4*. (D) Typical examples of CD31‐stained transversal sections. (E) Microvessel density (CD31+ area) tended increased in par‐AF and pers‐AF. (F) qPCR validated the upregulation of hypoxia‐induced transcription factors. (G) NFATC1 was found in the endocardium of pers‐AF patients. There were almost no signals found in non‐AF patients. ECM, extracellular matrix. Boxplots depict range and interquartile range

Endothelial cell proliferation, angiogenesis, and vascular endothelial cell signaling, characterized by processes, such as *regulation of vascular endothelial cell proliferation* and *regulation of vascular endothelial growth factor receptor signaling*, were among the continuously upregulated biological processes (Figure 4A). Correspondingly, endothelial cell gene expression (e.g., CD31 [*PECAM1*]*, ICAM1* or VE‐cadherin [*CDH5*]) and the expression of regulators of endothelial cell proliferation were increased (Figure 4B). The vascular endothelial growth factor receptor *(FLT1)* was the most significantly upregulated gene in pers‐AF versus non‐AF (FC = 0.90, FDR = 1.8^E−11^) (Figure 4C). The expression of *FLT1* and *CSPG4* was validated with qPCR (Figure 4C). Microvessel density, as a surrogate marker of angiogenesis, showed a tended increase in par‐AF and pers‐AF (Figure 4D,E).

Differentially expressed TFs driving angiogenesis included various increased endothelial Kruppel‐like factors (*KLF2, KLF4, KLF15*) and hypoxia‐induced factors (*HIF1A, HIF3A*; which were qPCR validated; Figure 4F).^15^, ^31^ Immunohistochemistry showed that NFATC1 could not only be observed in the epicardium (Figure 3E), but also in the endocardium of AF patients (Figure 4G). Almost no NFATC1 signals were detected in non‐AF patients and no NFATC1 signals were observed in interstitial endothelial cells. Furthermore, the majority of differentially expressed TFs discovered by GSEA of TF regulons were implicated in angiogenesis (*ETS1, ETS2, TP53*, myc‐associated factor X [*MAX*], *NFE2, ARNT/*HIF1β*, JUN*) (Figure S5).^31^, ^32^

### 3.5 Perivascular mesenchymal cells increase along with angiogenesis

**FIGURE 5:**
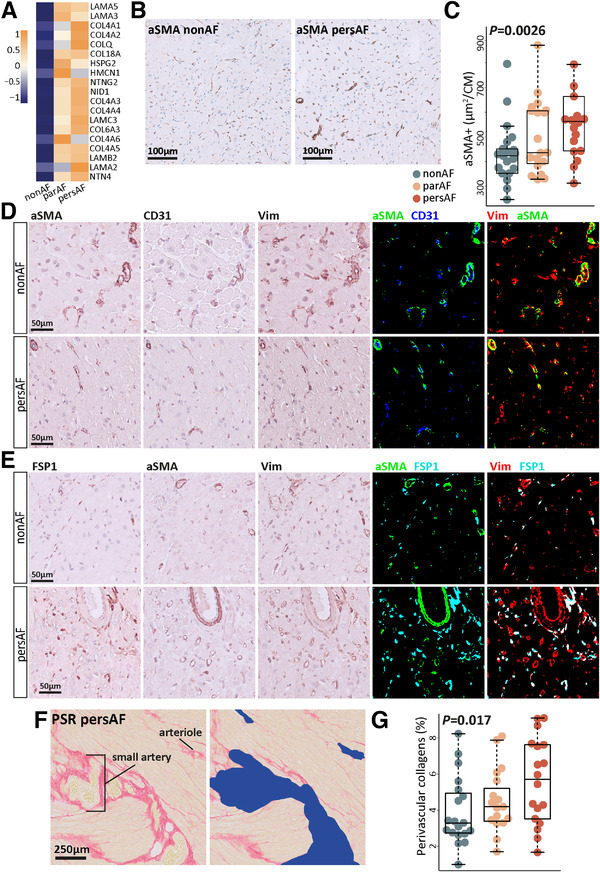
Perivascular structures increase in atrial fibrillation. (A) Heatmap showing the leading edge genes of the upregulated biological process *Naba basement membranes* (MSigDB). (B) Typical examples of αSMA‐stained transversal sections. (C) αSMA+ area fraction was increased in par‐AF and pers‐AF patients. (D) αSMA+ cells were mostly found surrounding small arterioles. Few lone αSMA+ myofibroblasts were identified. αSMA+ cells make up a proportion of vimentin+ cells. (E) αSMA+ cells and FSP1+ cells identified distinct cell types. FSP1+ cells make up a proportion of vimentin+ cells. (F) Perivascular collagen fraction was quantified using picrosirius red staining (yellow myocardium; red collagens). Vessels identified by their morphology were manually removed including perivascular collagens (blue right image). Perivascular fibrosis was defined as the difference in collagen (red) fraction between the left and right images. The myocardial area used for normalization was determined after large vessel removal (right image). (G) Perivascular fibrosis was increased in par‐AF and pers‐AF. Boxplots depict range and interquartile range

Along with angiogenesis, perivascular cell gene signatures were enhanced in par‐AF and pers‐AF patients compared to non‐AF patients (e.g., *PDGFRB, CSPG4, DLC1, PRKG1*).^33^, ^34^ The upregulation of perivascular cell signatures was accompanied by increased expression of genes that compose perivascular ECM structures such as laminins, non‐fibrillar collagens, perlecan, and nidogan (*LAMA2, LAMA5, LAMB2, LAMC3, COL4A1‐4, COL18A1, HSPG2, NID1*) (Figure 5A).^34^

With immunohistochemistry, an increase in perivascular αSMA‐positive cells was found in AF (Figure 5B,C). The αSMA‐positive cells were generally observed surrounding interstitial small arterioles (Figure 5D,E). FSP1‐positive and αSMA‐positive cells formed two distinct subgroups of vimentin‐positive cells (Figure 5D,E).^35^ Collagen content surrounding larger vessels was increased in AF (Figure 5F,G), suggesting that these myofibroblast‐like cells^35^ may contribute to ECM production.

### 3.6 Proteoglycan and glycoprotein synthesis is highly upregulated in AF

When looking at the components of ECM deposits, glycoprotein and proteoglycan synthesis were robustly found among the top upregulated biological processes (Figure 6A,B and Figure S6). Among the increased glycoproteins were tenascin‐C and thrombospondins (*TNC, THBS1, THBS2*) (Figure 6C). Furthermore, the gene expression of various proteoglycans, including versican and biglycan (*VCAN, BGN*), was increased (Figure 6C). The upregulation of proteoglycans was accompanied by increased gene expression of glycosaminoglycan (GAG) side chain synthesis (*CHSY1*), polymerization (*CHPF2*), transferase enzymes (*CHST1, CHST2, CHST3, CHST6, CHST7, CHST11; HS3ST2, HS3ST3B1*), hyaluronic acid synthase (*HAS2*), and hyaluronic and proteoglycan link proteins (*HAPLN1, HAPLN2, HAPLN3*) (Figure S6).

FIGURE 6Proteoglycan and glycoprotein expression is increased in atrial fibrillation. (A) The top 15 upregulated biological processes in the comparison pers‐AF versus non‐AF. Bold, processes are directly related to ECM production. (B) DEG included in the upregulated biological processes *ECM proteoglycans* (Reactome) and *Naba proteoglycans* (MSigDB). The largest fold changes can be found between par‐AF versus non‐AF. (C) qPCR validated increased expression of glycoproteins and proteoglycans in par‐AF and pers‐AF in the study cohort and an independent cohort. (D) Typical examples of interstitial glycosaminoglycans (GAGs) visualized with alcian blue. Contrasts were enhanced for visualization only. (E) GAGs showed an increase in par‐AF and pers‐AF. (F) Typical examples of alcian blue stainings showing that GAGs were most abundant in the endocardium and myocardium adjacent to the endocardium, and fewer proteoglycans were observed in or toward the epicardium. Contrasts were enhanced for visualization only. (G) The hyaluronic acid receptor CD44 could be seen in the epicardial basal layer, the fibrotic subepicardial layer, the endocardium, and surrounding small arterioles. There was no clear difference in CD44 between non‐AF and AF patients. (H and I) Quantification of interstitial collagens on picrosirius red stainings found increased interstitial collagen fractions in par‐AF and pers‐AF patients. AB, alcian blue; EAT, epicardial adipose tissue; PSR, picrosirius red. Boxplots depict range and interquartile range

**Figure:**
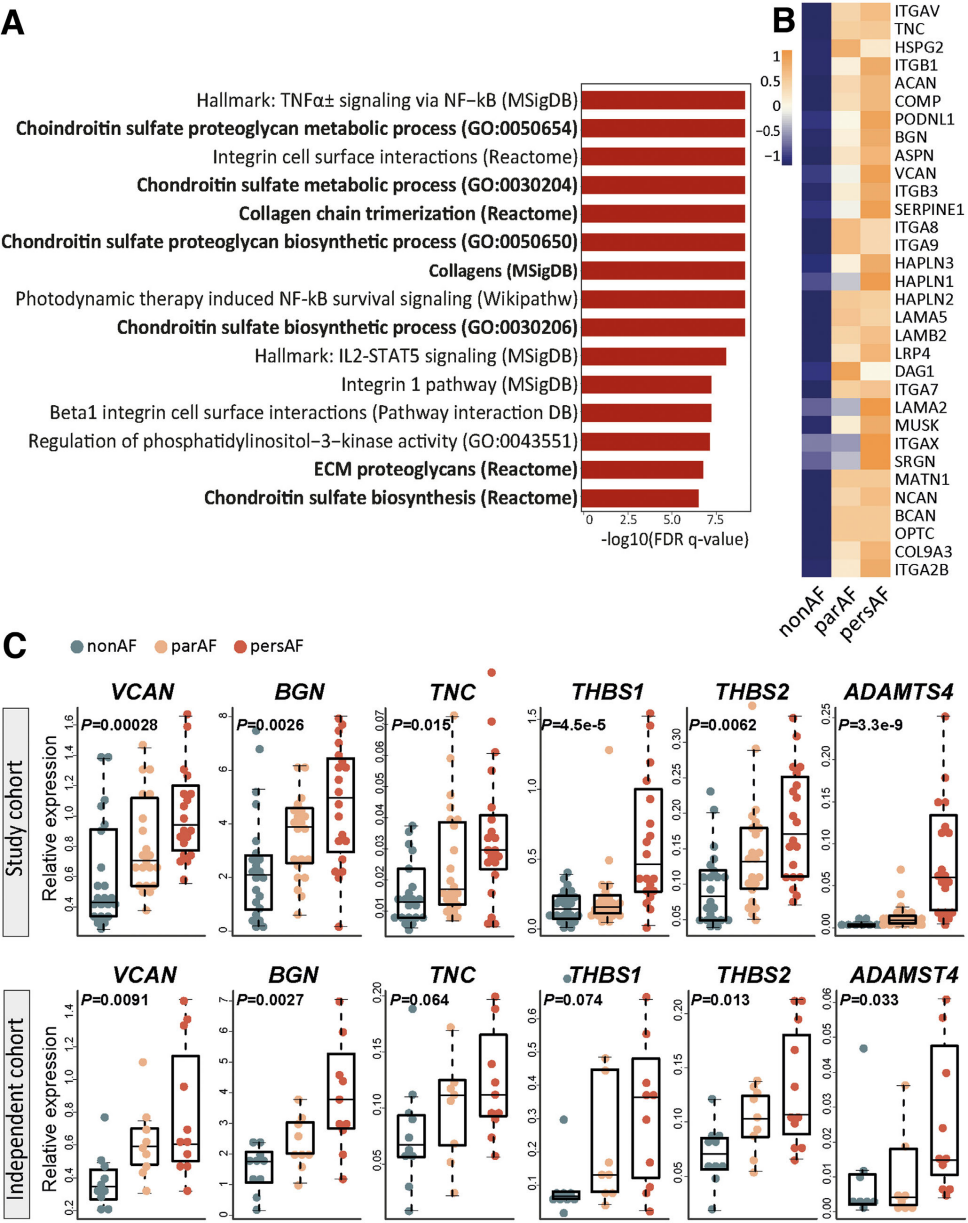

**Figure:**
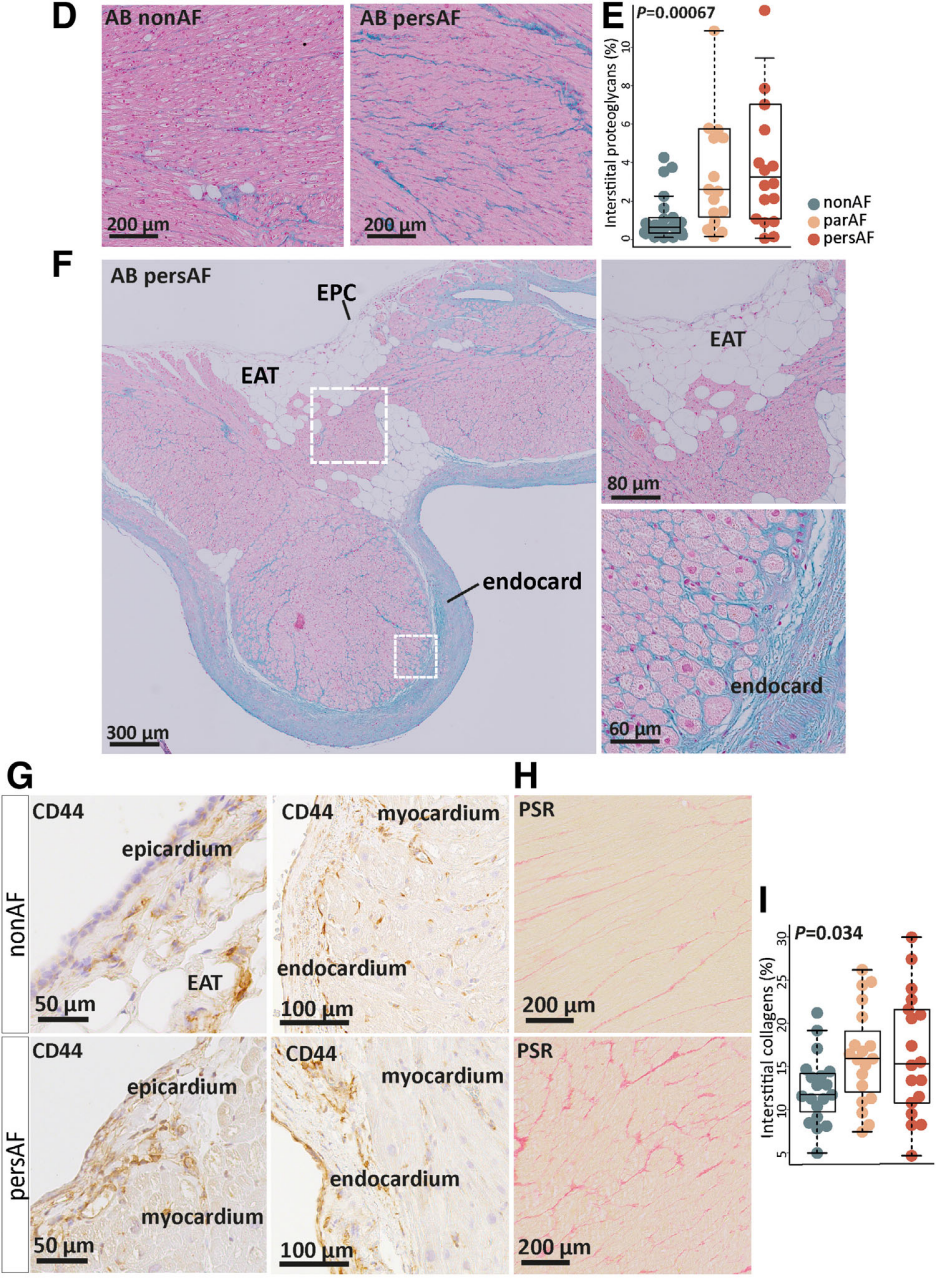

The increased gene expression of glycoproteins and proteoglycans was validated with qPCR in the same and in an independent cohort (Figure 6C). Atrial tissue sections stained with alcian blue corroborated the increase of interstitial GAGs in AF (Figure 6D,E). GAGs were most abundant in the endocardium and myocardium adjacent to the endocardium in 88%–91% of the examined atria, while fewer proteoglycans were observed in or toward the epicardium (Figure 6F). Cells expressing the hyaluronic acid receptor CD44 were observed in non‐AF and AF patients in the epicardial basal layer, the subepicardial fibrotic layer, as well as in the endocardial layer and perivascular niche (Figure 6G).^36^

Collagen biosynthesis was also upregulated, characterized by increased gene expression of fibrillary collagens (e.g., *COL16A1, COL21A1, COL9A3*) and basement membrane collagens (*COL4A1‐4, COL18A1*) in AF (Figure S5). In parallel, genes such as prolyl‐hydroxylases and cross‐linking lysil oxidases (*P3H3, LOXL1, LOXL2*) were increased in AF (Figure S5). The expression of type I and type III collagen (*COL1A1*, *COL3A1*) genes was only modestly and nonsignificantly increased in AF (Figure S5), but atrial tissue sections stained with picrosirius red showed an increase in interstitial collagen content (Figure 6H,I).

In addition to ECM gene upregulation, the expression of ECM degrading constituents was increased in par‐AF and pers‐AF, such as hyaluronidases (*HYAL1, HYAL2, HYAL4*), and a disintegrin and metalloproteinase with thrombospondin motifs (ADAMTS) families (Figure S5). *ADAMTS4* and *ADAMTS9* were among the most significantly increased genes (FC = 4.1 and 1.74, FDR = 2.1^E−9^ and 7.5^E−7^ respectively; Figure 6C and Figure S6). Several matrix metalloproteinases (MMP), including *MMP9, MMP19*, and *MMP25* were increased and some (e.g., *MMP14, MMP24*, and *MMP28*) were decreased. At the same time, the tissue inhibitor of metalloproteinase 1 (*TIMP1*) was found downregulated. Together, these data suggest a wide‐ranging increase of ECM turnover.

### 3.7 Interconnecting signaling

**FIGURE 7:**
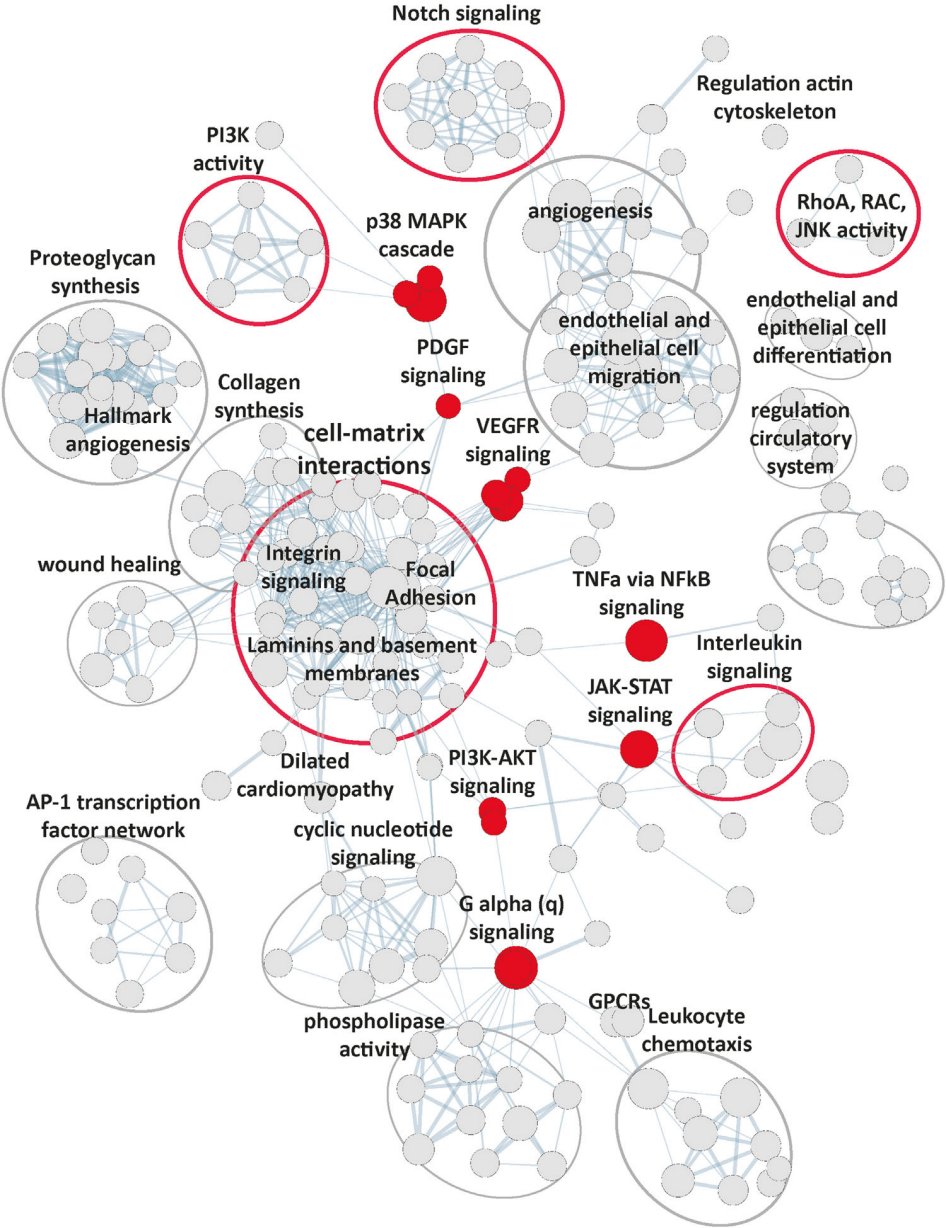
Interconnecting signaling. Enrichment map showing all upregulated (FDR *q*‐value <.05) biological processes discovered by gene set enrichment analysis (pers‐AF vs. non‐AF; Supporting Data File). Cell–matrix interactions can be found interconnecting various processes. Highlighted (red) are the signaling pathways directly related to structural remodeling processes

EMT, angiogenesis, and ECM genes were interconnected through signaling pathways and feedback systems with cell–matrix interactions at the center of connectivity (Figure 7 and Figures S7–S9). Integrins in particular demonstrated a broad upregulation (*ITGA1, ITGA2B, ITGA5* till *ITGA10, ITGAV, ITGAX, ITGB1, ITGB3*), and intracellular integrin signaling pathways were upregulated such as *integrin signaling, integrin cell surface interactions*, and *integrins in angiogenesis* (Figure S7).^37^

With respect to processes related to cytoskeleton reorganization, regulation of actin cytoskeleton reorganization, regulation of RhoA activity, and regulation of RAC1 activity were upregulated (e.g., PLEKHG2, DOCK6, PREX1, ARAP3, DLC1, ROCK1) (Figure 7).

Several processes related to PI3K activity were upregulated, demonstrated by the numerous processes related to PI3K signaling (Figure 7). Moreover, Class IA *PIK3R3* regulatory subunit was among the most significantly increased genes (FC = 1.17, FDR = 6.77^E−10^; qPCR validated; Figure S10). Besides, Class IB *PIK3R5*, Class II *PIK3C2A*, and *AKT1* were upregulated. Figure 7 and Figure S9 show that PI3K activity can be activated by integrin signaling and growth factors and in turn can activate EMT and angiogenesis.^15^ Receptor tyrosine kinases (RTKs) signaling such as VEGF signaling and PDGF signaling (Figure 7) were upregulated along with the upregulation of various growth factors and their receptors (*PDGFB, PDGFRB, PDGFRA, VEGFA* and the VEGF receptor *FLT1*; Figure 4C).

Figure 7 displays various other upregulated signaling pathways that relate to EMT and ECM remodeling including *NOTCH signaling pathway* (e.g., *NOTCH1, NOTCH3, HEYL, HEY1*). Furthermore, multiple proinflammatory pathways were upregulated, including cytokines and immune cells such as *TNFa via NFkB signaling* (e.g., *KLF9, SERPINE1, TNFAIP3, TRAF1*), *interleukin signaling* (e.g., *IL6, IL4R, IL1R2, CSF3R*), and *leukocyte chemotaxis* (e.g., *CXCR1, CXCR4, CXCL2)*.

Detailed information regarding the individual nodes and complex signaling that gave rise to Figure 7 and the leading edge genes of each node can be found in the Supporting Data File.

## 4 DISCUSSION

We describe a contextual framework of structural remodeling in human AF, depicting a highly dynamic system involving EMT, endothelial cell proliferation, and ECM remodeling (summarized in Graphical Abstract). Transcriptome sequencing identified gene expression signatures that predominantly followed an ordinal trend from no to paroxysmal to persistent AF. These continuous processes of AF were the focus of this study. We found a downregulation of epicardial cell markers and cell–cell junction genes in AF in contrast to an increase in mesenchymal cell gene expression and an upregulation of biological processes related to EMT. EMT was supported by immunohistochemistry showing changes in EMT regulatory TFs such as SNAIL and TWIST, and disorganization and thickening of the epicardial monolayer in AF patients. Within the subepicardium of AF patients, there were less MSC cells, whereas the interstitial MSC proportion increased. These observations may indicate MSC migration from the subepicardium into the myocardium.^14^ Meanwhile, endothelial cell marker gene expression increased along with processes related to angiogenesis and microvessel density. Perivascular (myo)fibroblast‐like αSMA‐positive cells and FSP1‐positive cells were increased in AF, which may have contributed, at least partially, to the increased interstitial vimentin‐positive MSC fraction in AF. These changes in the proportions of MSCs were associated with increased expression of genes involved in ECM synthesis and degradation. The increased expression of glycoproteins and proteoglycans together with increased interstitial GAGs suggested ECM remodeling beyond increased structural collagen deposits. Various pathways known to regulate both EMT and endothelial cell proliferation were upregulated, including integrin signaling, Notch signaling, RTK signaling, PI3K signaling, and inflammatory cytokines.^15^ These pathways can induce *SNAI1* and *ZEB1* expression, consistent with both EMT and angiogensisis.^15^ This study does not provide functional data, and further in vitro and in vivo studies are needed to verify our findings. The presented data may therefore be used to design such further studies as we demonstrate how, in multifactorial human atrial remodeling, multiple processes involved in AF structural remodeling may coexist and possibly enhance one‐another.

Decreased EPC gene expression and epicardial morphological changes imply re‐activation of the epicardial layer in AF. We lately reported that signs of atrial EMT may even precede the first occurrence of incident AF.^6^ The re‐activation of quiescent epicardial cells has long been recognized in lower vertebrates such as zebrafish^38^ and has been described in the setting of acute myocardial infarction.^39^ However, EPC re‐activation was only recently reported in association with fibro‐fatty infiltration in mitral valve disease‐related AF.^14^ Suffee et al. functionally investigated specific EMT regulatory pathways involving angiotensin II and atrial natriuretic peptide.^14^ These pathways led to the differentiation of EPCs into myofibroblasts and epicardial adipocytes, respectively. Our study shows that multiple pathways are involved in EMT in human AF, which may function in parallel or synergistically. These findings are supported by multiple differentially expressed regulatory TFs such as *SNAI1* and *ZEB1*, and by upregulated signaling pathways such as Notch, PI3K‐AKT, and cell–matrix interactions.^15^

Contrary to the findings of Suffee et al., we did not find increased numbers of confined interstitial myofibroblasts.^14^ Instead, various fibroblast‐like cell fractions and perivascular cell fractions were found increased in AF. We also did not find a statistical association between EMT and increased epicardial fat deposits,^14^ even though an association between increased EAT volumes and AF severity has been demonstrated by CT imaging.^40^ A statistical difference in EAT area may have been attenuated, as the EAT area in pers‐AF patients was either very large or very small. Small EAT areas may have been caused by fibrotic replacement of EAT, which may enhance transmural conduction heterogeneity.^41^, ^42^ The differences between our study and Suffee et al. may also relate to the patient cohorts and the source of atrial tissues studied. Opposed to Suffee et al., who studied right atria, we investigated left atrial appendages. Although there are known differences at the molecular level between left and right atrial tissues,^43^ these differences appear limited when investigating, for example fibrosis, histologically.^44^

We found endothelial cell proliferation, angiogenesis, and microvessel density continuously upregulated along the comparisons of no, paroxysmal, and persistent AF, corresponding to a previous report.^45^ Angiogenesis has been explored extensively in cardiac hypertrophy and heart failure, but its role in AF is less well established.^31^ Here, we cannot determine cause and effect, but suggest that angiogenesis results from increased energy and oxygen demands due to the tachyarrhythmia or results from pathology, such as fibrosis, interfering with oxygen diffusion.^31^ Apparently, the increased microvessel density in AF patients may be insufficient to assure adequate perfusion.^31^ Hypoxic endothelial cells may support structural remodeling by pro‐fibrotic RTK signaling or by the secretion of factors such as interleukins or *LOXL2* in exosomes.^46^

Persistent tissue hypoxia, implicated in this study by increased *HIF1A* and *HIF3A* gene expression, may drive EMT and endothelial cell proliferation and differentiation.^15^, ^31^ Endothelial cells may undergo phenotypic conversion mediated by TFs such as *HIF1A, HIF3A, ZEB1*, or *SNAI1* and obtain (myo)fibroblasts‐like phenotypes and thereby contribute to structural remodeling.^46^ Zeisberg et al. studied heart failure in mice and reported that endothelial to mesenchymal transition (EndMT) contributes to the pool of αSMA‐positive or FSP1‐postive fibroblast‐like cells by 27%–35%.^35^ Further research is needed to specify subtypes of interstitial (myo)fibroblast‐like cells, as markers (e.g., FSP1) are not mutually exclusive.^11^ Nevertheless, the increase in perivascular cells in this study suggests that EndMT may be an ongoing process in AF. Contradicting ongoing EndMT was an observed increase in endothelial cell marker gene expression and microvessel density, whereas EndMT is characterized by decreased endothelial cell gene expression. This suggests that if EndMT is ongoing, endothelial cell proliferation outpaces differentiation from the endothelial‐cell phenotype.

The changes in epicardial, endothelial, and perivascular cell gene expression signatures in our study characterize the plasticity of MSC.^15^ In injured zebrafish and mice hearts, epicardial‐derived cells demonstrated to differentiate fibroblasts, perivascular cells, smooth muscle cells, and adipocytes. Epicardial‐derived cells were found to modulate ECM deposits and paracrine signaling, which in turn regulate angiogenesis and cardiomyocyte survival and proliferation.^38^, ^47^

Here, we found indications for re‐activation of MSC in the epicardial monolayer and possibly the perivascular niche.^35^, ^48^ The source of fibroblasts involved in atrial structural remodeling is still debated. The presence of both epicardial re‐activation and endothelial cell proliferation suggests extensive, transmural MSC differentiation and activation. Migration of these cells was suggested by redistribution of MSC from the subepicardium to the myocardial interstitium and was supported by upregulated Rho GTPase signaling, which is involved in myofibroblasts activation and cytoskeleton reorganization and cell movement.^15^ Migration was furthermore supported by enhanced expression of ECM degradation genes such as MMPs that allows invasive behavior, and by the broad upregulation of integrins, which is consistent with PI3K‐coordinated assembly of cell–matrix adhesions at the leading edge.^15^ Our results suggest that the MSC response in AF is not limited to the activation of a single pathway in either the epicardium or endocardium, but includes activation, proliferation, and migration of various cell phenotypes throughout the atrium.

Angiogenesis, EndMT, and EMT are regulated by overlapping TFs and pathways with significant cross‐talk and feedback mechanisms (e.g., *SNAI1, HIF1A, HIF3A*, Notch signaling, inflammation).^15^, ^31^ In addition, most of the discovered upregulated (signaling) pathways and biological processes can directly be interconnected. For example, PI3K‐AKT can be considered a regulatory hub: it can be activated by several upregulated pathways such as VEGF or PDGF signaling and can initiate multiple downstream pathways that were upregulated such as the p38/MAPK and JNK pathways. MAPK can increase the expression of EMT TFs and regulators of migrations such as Rho GTPases and JNK.^15^ Indeed, inhibition of PI3K‐AKT signaling has been shown to inhibit EMT in cancer cells.^15^, ^49^ TGF‐β signaling is another key activator of EMT and EndMT.^15^, ^16^ We describe multiple signaling pathways that can be activated by TGF‐β, including PI3K‐AKT signaling.^15^ However, we did not find TGF‐β (signaling)‐related genes to be clearly up‐ or downregulated. The pathways we describe, however, can also be activated independently from TGF‐β.^15^, ^16^ Nevertheless, a role for TGF‐β at the protein level is possible, especially as we found several proteoglycans upregulated known for their ability to store TGF‐β (e.g., *LTBP1, LTBP2)*.^50^, ^51^

The production of ECM components was highly upregulated in this study and encompassed more than increased structural collagen deposits alone. We found an increased expression of glycoprotein and proteoglycan genes and demonstrated for the first time an interstitial increase of GAGs (alcian blue) in AF. Increased GAGs in AF were predominantly found toward the endocardium, whereas collagens increased in the interstitium, perivascularly, and in the subepicardial layer. We recently reported that an upregulation of glycoproteins and proteoglycans may even precede the first onset of AF.^6^ These findings suggest that changes in ECM composition and morphology may be an early, dominant feature of ECM remodeling in AF.

It needs to be determined how glycoproteins and proteoglycans could contribute to increased arrhythmogenicity. Glycoproteins and proteoglycans can modify an inflammatory response, mesenchymal cell proliferation, migration, and signaling.^8^, ^52^ In failing human hearts, chondroitin sulfate glycosaminoglycans were found accumulated and regulated inflammation and fibrosis. Modification of chondroitin sulfate chains with recombinant human arylsulfatase B attenuated cardiac fibrosis.^53^

Proteoglycans are present in MSC niches and may play an important regulatory role in EMT. For example, the hyaluronic acid receptor CD44, which was found to activate EMT after injury in zebrafish,^36^ was found in the epicardium, endocardium, and perivascular niche. Proteoglycans may connect to the actin cytoskeleton of epithelial and endothelial cells and initiate apical–basal polarity changes, followed by actin cytoskeleton re‐arrangements and migration.^15^, ^37^ Thrombospondins may initiate antiangiogenic signaling when binding to cell integrins,^54^ and basement membrane heparin sulfate proteoglycans (*HSPG2*, *NID1, COL18A1*) can function as both pro‐ and anti‐angiogenic factors by binding to and regulating the amount of growth factors in proximity to cell‐surface receptors.^55^

Overall, the complex nature of the discovered processes precludes us from pointing at one specific regulatory network or one specific origin of MSCs. mRNA sequencing of bulk tissue does not provide data regarding miRNA regulation, single‐cell gene expression, or protein expression. EMT is a process that is significantly regulated by miRNAs.^56^, ^57^ MiRNA co‐sequencing could provide insight into the role of miRNA regulation of EMT. The data presented in this study is cross‐sectional, and we can therefore only speculate about temporal changes in gene expression, such as a decrease of *WT1* expression being the result of complex balanced signaling or completed EMT. It is important to note that the data presented are associative only and due to the aforementioned spatiotemporal regulation of the transcriptome, gene expression changes are not always equivalent to functional changes. For a full understanding of the physiology underlying structural remodeling in AF, it is essential that our findings are confirmed in functional models, including epicardial cell cultures of non‐AF and AF patients, to study the relevant signaling pathways and cellular response (e.g., migration and ECM production) upon stimulation.^58^ Furthermore, lineage tracings in mice exposed to an AF stressor like angiotensin‐II could tell us the detailed origin of the increased MSC population upon AF pathogenesis and establish proof of the simultaneous existence of both EMT and EndMT.^14^ Animal studies further have the ability to determine a time‐relation between EMT and AF vulnerability. Despite the lack of such functional studies, our data provide a high‐resolution description of the AF transcriptome, which may provide the necessary cues for future uncovering of the multifactorial mechanisms of structural remodeling in AF.

## 5 CONCLUSION

Our mostly descriptive data allowed us to draw up a contextual framework for structural remodeling in human AF. Our study provides evidence demonstrating that human structural remodeling in AF involves a mesenchymal cell response that arises from the epicardium and perivascular niche through EMT and involves endothelial cell proliferation and differentiation. The mesenchymal cell activation was not the result of a single activated regulatory pathway. Resulting (myo)fibroblast‐like cells may provide paracrine signaling and may orchestrate the complex structural remodeling in AF, which encompasses not only collagen biosynthesis but also glycoprotein, proteoglycan, and glycosaminoglycan deposits. Our results suggest that EMT and endothelial cell proliferation work in concert and characterize (myo)fibroblast recruitment and ECM remodeling. These results may help guide future functional research toward the discovery of targets for AF therapies.

## Supporting information

SUPPORTING INFORMATION

SUPPORTING INFORMATION

## Data Availability

The transcriptome sequencing data that supported the current study are available under EGAS00001005295. All data will be made available upon reasonable request to the corresponding author.
