# Supplementary material for: Epicardial and endothelial cell activation concurs with extracellular matrix remodeling in atrial fibrillation
Source: Clin Transl Med. 2021 Nov 4;11(11):e558. doi: 10.1002/ctm2.558 (PMC8567047; doi:10.1002/ctm2.558)
Supplement: Supplementary file 2 — SUPPORTING INFORMATION [file CTM2-11-e558-s002.docx]

**SUPPLEMENTARY MATERIALS**

**Epicardial and endothelial cell activation concurs with extracellular matrix remodelling in atrial fibrillation**

Nicoline W.E. van den Berg, MD*, Makiri Kawasaki, PhD*, Benedetta Fabrizi, MSc*, Fransisca A. Nariswari, MSc*, Arianne C. Verduijn, BSc*, Jolien Neefs, MD, PhD*, Robin Wesselink, MSc*, Rushd F.M. Al-Shama, MD*, Allard C. van der Wal, MD, PhD¥, Onno J. de Boer, PhD¥, Jan Aten, PhD¥, Antoine H.G. Driessen, MD PhD*, Aldo Jongejan, PhD‡, Joris R. de Groot, MD, PhD*

* Amsterdam UMC, University of Amsterdam, Heart Center; department of Clinical and Experimental Cardiology, Amsterdam Cardiovascular Sciences, Meibergdreef 9, Amsterdam, The Netherlands

¥ Amsterdam UMC, University of Amsterdam; department of Clinical Pathology, Meibergdreef 9, Amsterdam, The Netherlands

‡ Amsterdam UMC, University of Amsterdam, Department of Epidemiology & Data Science, Meibergdreef 9, Amsterdam, The Netherlands

**Supplementary Materials and Methods**

**Library preparation**

Libraries were prepared from 250ng of total RNA after rRNA depletion with QIAseq FastSelect (Qiagen). Strand-specific sequencing libraries were prepared using rRNA depletion with the NEBNext rRNA Depletion kit. cDNA synthesis was performed with the NEBNext RNA First Strand Synthesis, NEBNext Ultra Directional RNA Second Strand Synthesis Modules, NEBNext Directional RNA Library Prep Kit for Illumina and the NEBNext Ultra II DNA Library Prep Kit for Illumina (New England BioLabs). Library quantification was performed with the Kapa Illumina GA Revised Primers-SYBR Fast Universal kit (Kapa Biosystems). Average size fragment were determined (LabChip GX, PerkinElmer).

**RNA sequencing data analyses**

Reads were mapped towards the human reference genome (GRCh38v94) using HiSAT2 v2.1.0 with default settings. Uniquely mapped reads were counted with HTSeq v0.11 11 ("-m union -f bam -r name -s reverse -a 10 -t exon -i gene_id"). Genes with zero counts in all samples were removed before further analysis. Genes were filtered out that showed a minimum expression of  1 counts per million in 20 or more samples. Normalization of read counts was performed using the Trim mean of M-values (TMM) methods (R package EdgeR). Hierarchical clustering of the DE genes was performed using a Pearson correlation coefficient and average linkage for sample clustering and Spearman’s rank correlation coefficient with average linkage for gene clustering. Hierarchical clustering of patients was performed completely unsupervised and unsupervised per study group. Mean CPM were used to summarize group expression values used for expression heatmaps.

**Supplementary Tables**

**Supplementary Table S1. Patient characteristics of independent validation cohort**

|  | **All patients**  **n=30** | **nonAF**  **n=10** | **ParAF**  **n=9** | **PersAF**  **n=11** |
| --- | --- | --- | --- | --- |
| Sex, female, n(%) | 6 (5) | 0 | 4 (44.4) | 2 (18.2) |
| Age, years (±SD) | 65.5 ±5.4 | 67.9 ±4.1 | 66.1 ±6.0 | 62.9 ±5.4 |
| BMI, kg/m^2^ (±SD) | 27.2 ±3.2 | 26.3 ±2.0 | 27.3 ±4.6 | 27.8 ±3.0 |
| Myocardial infarction, n(%) | 5 (16.7) | 4 (40) | 0 | 1 (9.1) |
| PCI, n(%) | 5 (16.7) | 3 (30) | 1 (11.1) | 1 (9.1) |
| CHA_2_DS_2_-VASc [IQs] | 2 [1-3] | 2 [2-2.75] | 3 [1-3] | 1 [0.5-2] |
| CHA_2_DS_2_-VASc ≥4, n(%) | 3 (10) | 1 (10) | 2 (22.2) | 0 |
| Vascular disease, n(%) | 14 (46.7) | 10 | 2 (22.2) | 2 (18.2) |
| Hypertension, n(%) | 11 (36.7) | 2 (20) | 4 (44.4) | 5 (45.5) |
| Diabetes Mellitus, n(%) | 4 (13.3) | 3 (30) | 1 (11.1) | 0 |
| Congestive heart failure, n(%) | 1 (3.3) | 0 | 1 (11.1) | 0 |
| Stroke/TIA/embolus, n(%) | 1 (3.3) | 0 | 1 (11.1) | 0 |
|  |  |  |  |  |
| **Echocardiography** |  |  |  |  |
| Max LAVI, ml/m^2^ (±SD) | 35.1 ±10.9 | 25.1 ±6.4 | 38.8 ±10.6 | 42.4 ±7.2 |
| LVEF, % (±SD) | 50.8 ±11.0 | 47.0 ±9.1 | 56.3 ±5.1 | 47.3 ±13.4 |
|  |  |  |  |  |
| **Medication** |  |  |  |  |
| Antiplatelet, n(%) | 10 (33.3) | 10 | 0 | 0 |
| Anticoagulation, n(%) | 19 (63.3) | 0 | 8 (88.8) | 11 |
| ACE inhibitors, n(%) | 6 (20) | 1 (10) | 3 (33.3) | 2 (18.2) |
| Angiotensin receptor blockers, n(%) | 4 (13.3) | 1 (10) | 1 (11.1) | 2 (18.2) |
| Class IA AAD | 0 | 0 | 0 | 0 |
| Class IC AAD | 4 (13.3) | 0 | 3 (33.3) | 1 (9.1) |
| Class II AAD | 16 (53.3) | 8 (80) | 2 (22.2) | 6 (54.5) |
| Class III AAD | 12 (40) | 0 | 6 (66.6) | 6 (54.5) |
| Class IV AAD | 2 (6.7) | 0 | 0 | 2 (18.2) |
| Digoxine, n(%) | 3 (10) | 0 | 1 (11.1) | 2 (18.2) |

Abbreviations: AAD, antiarrhythmic drugs; ACE, angiotensin converting enzyme; AF, atrial fibrillation; BMI, body mass index; IQ, interquartiles; LAVI, left atrial volume index; LVEF, left ventricular ejection fraction; PCI, percutaneous coronary intervention; PVI, pulmonary vein isolation; SD, standard deviation; TIA, transient ischemic attack

**Supplementary Table S2. Primers for real-time PCR quantification**

| **Gene** | **Forward 5’-3’** | **Reverse 5’-3’** |
| --- | --- | --- |
| *POLR2A* | CGCATCATGAACAGCGATGA | TGTACACCTTGCTGATCTGCTC |
| *HPRT* | TGACACTGGCAAAACAATGCA | GGTCCTTTTCACCAGCAAGCT |
| *PGK1* | CGACCCTTCCTGGCCATC | GTGCCAATCTCCATGTTGTTGAG |
| *GUSB* | GCGTCCCACCTAGAATCTGC | ATCCACATACGGAGCCCCC |
| *RPL32* | ACGTCAAGGAGCTGGAAGTG | GGGGTTGGTGACTCTGATGG |
| *ADAMTS4* | GGTCAAGGTCCCATGTGCAAC | GAATGCGGCCATCTTGTCATC |
| *BGN* | GCTGTACAGGCTGGGC | GTCGTTGACACCCACTTTGG |
| *COL1A1* | CTGAGCCAGCAGATCGAGAA | ATCCAGTACTCTCCACTCTTCCA |
| *COL3A1* | CCTGAAGCTGATGGGGTCAA | CAGTGTGTTTCGTGCAACCAT |
| *FN1* | CTGCAAGCCCATAGCTGAGA | GAAGTGCAAGTGATGCGTCC |
| *VCAN* | AAGACAGGACCTGATCGCTGC | GTGGCTCCATTACGACAGGG |
| *THBS1* | TGAGTGTCACTGTCAGAACTCAG | GACCATGGAGACCAGCCATC |
| *THBS2* | ATTCGCCATCAAGGCAAGGA | CGTCAAAACCTACAGCGATGC |
| *ITLN2* | CACTGAGCATCACTGCATCG | GAGAAGTCCCCACACTGACG |
| *WT1* | ACCAAAGGAGACATACAGGTGTGAA | ACCGACAGCTGAAGGGCTTT |
| *KRT19* | GCTGAGCATGAAAGCTGCCT | GCTCACTATCAGCTCGCACA |
| *SERPINE1* | CACGAGTCTTTCAGACCAAGAG | GTCCATGATGATCTCCTCGGG |
| *TNC* | TACACAGGCGAGAAAGTGCC | AGTGTGTATTCCGTGGCAGG |
| *HIF1A* | GGATGCTTGCCAAAAGAGGTG | AGGGAGAAAATCAAGTCGTGC |
| *HIF3A* | AGTCCTCTTTCGGCTGATCT | CAGCATCTCCAAATCCAGAGC |
| *FLT1* | GAAGGGAGCTCGTCATTCCC | AGCCCTTTCTACTGTCCCAGA |
| *PIK3R3* | CGATTTCGCAGAGAGGGGAA | AGCCAGACATTCAGGCGTTT |
| *CSPG4* | CTTCACTCAGGCAGAGGTCTA | GGACAGGCAGCCTCAAAAGA |

**Supplementary Figures**

**Supplementary Figure S1. Unsupervised hierarchical clustering separates nonAF from persistent AF.**

**
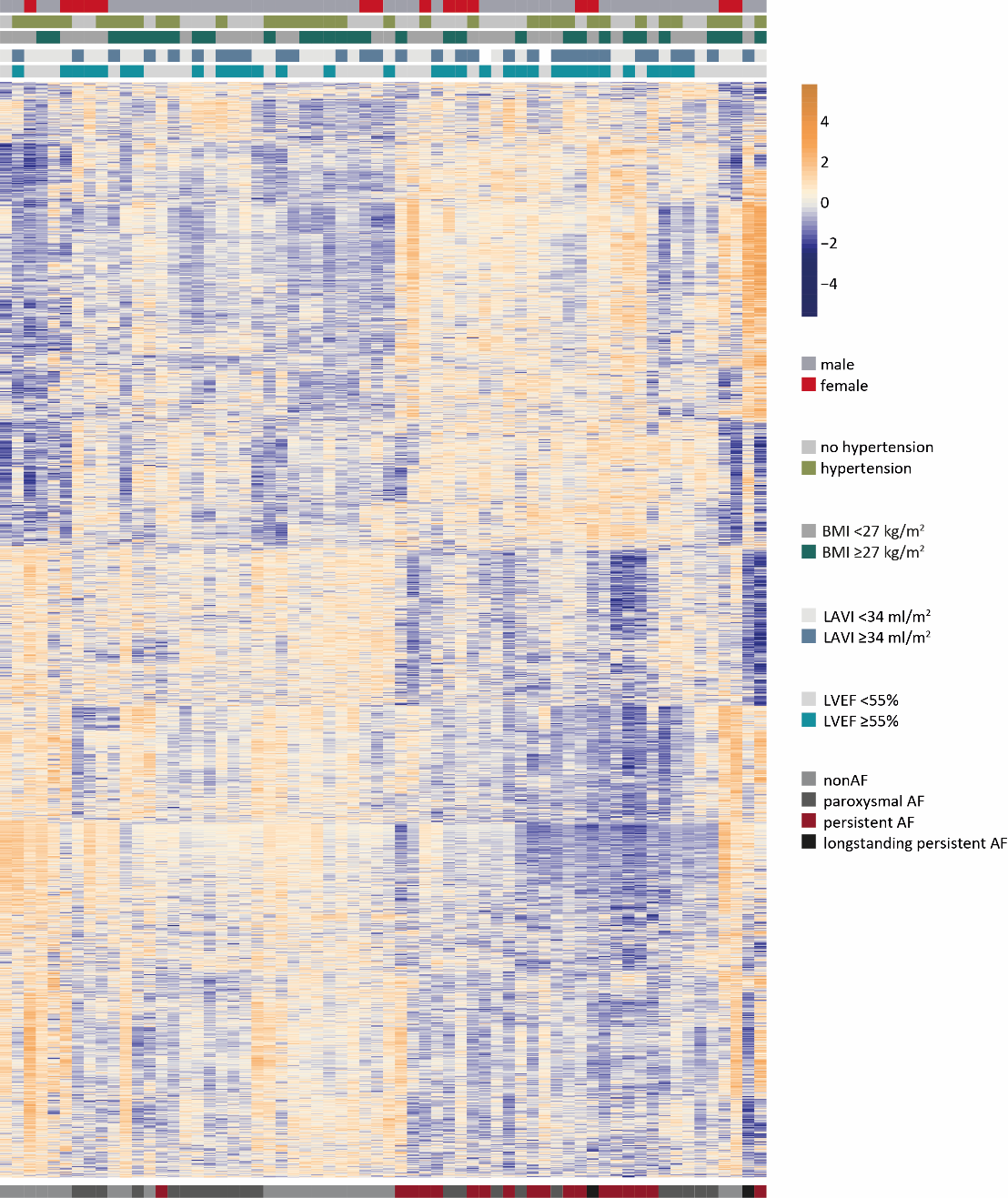
**

**Supplementary Figure S2. Expression plots of EMT transcription factors.**

**
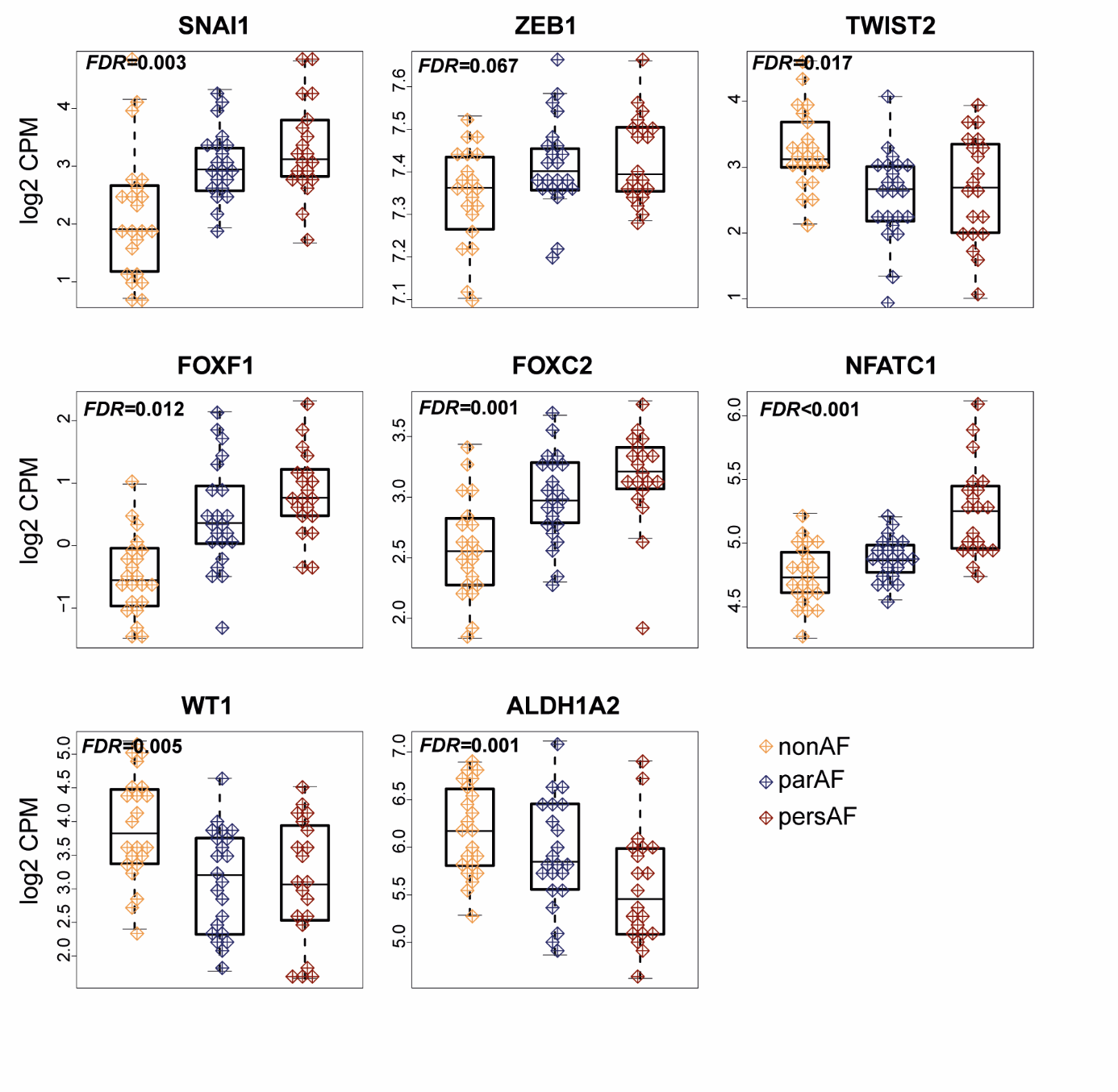
**

**Supplementary Figure S3. Histological quantification of EAT area reveals no difference between AF and nonAF**


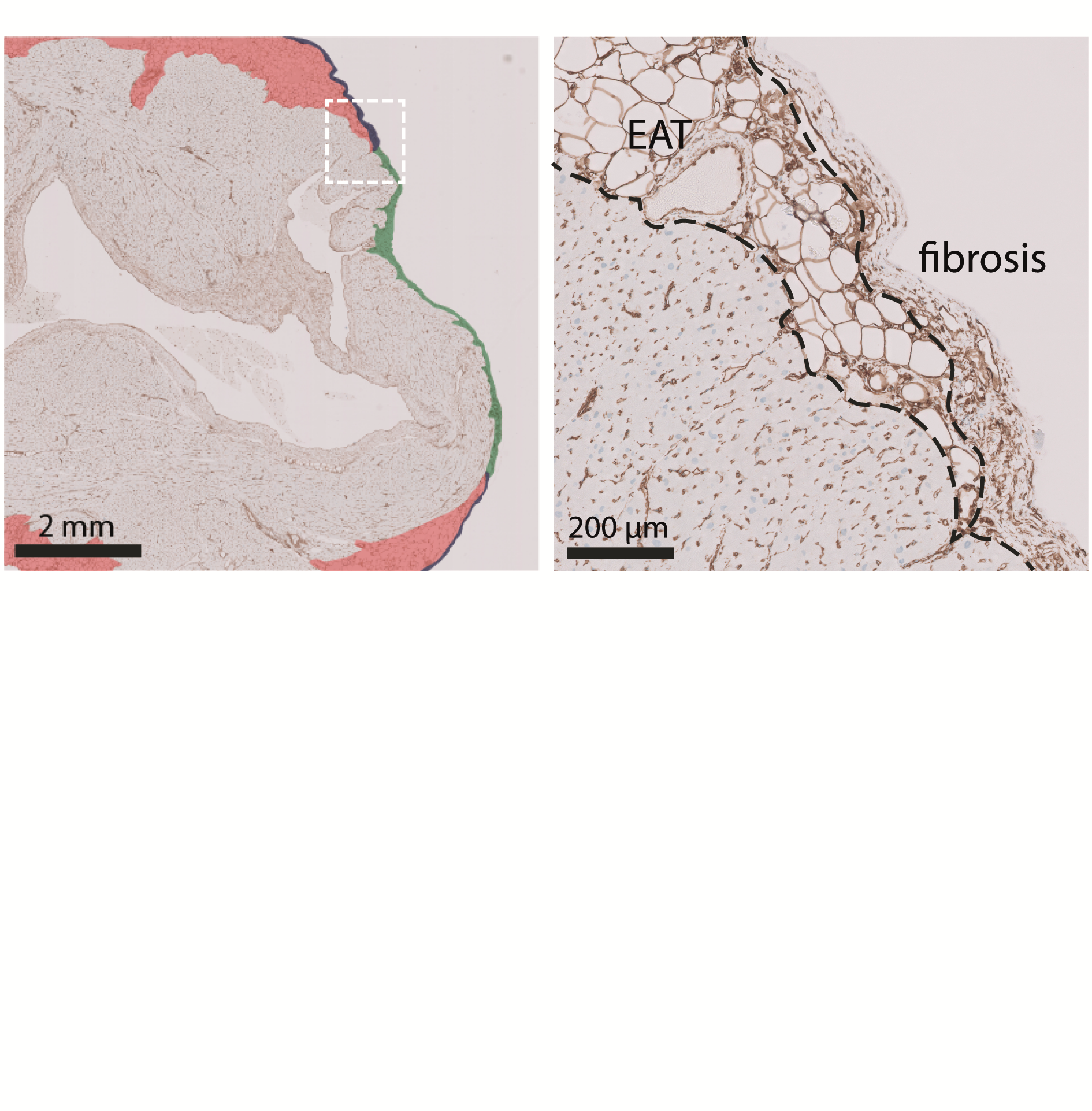


**Supplementary Figure S4. NFATc1 was not present in interstitial (endothelial) cells.**

**
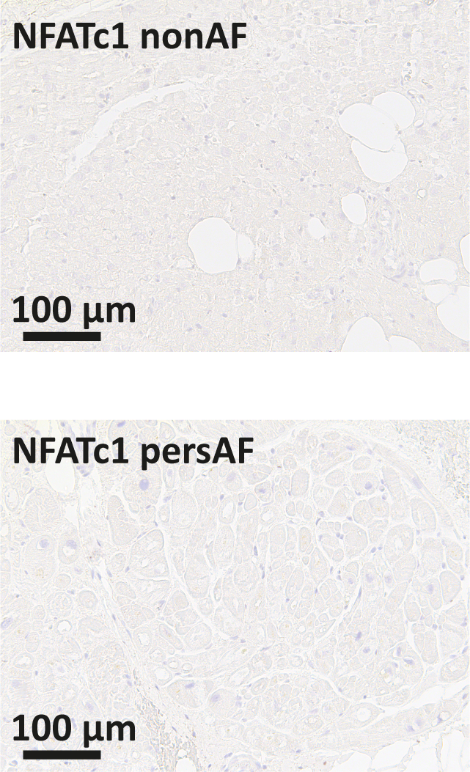
**

**Supplementary Figure S5. Transcription factors associated with angiogenesis.**

**
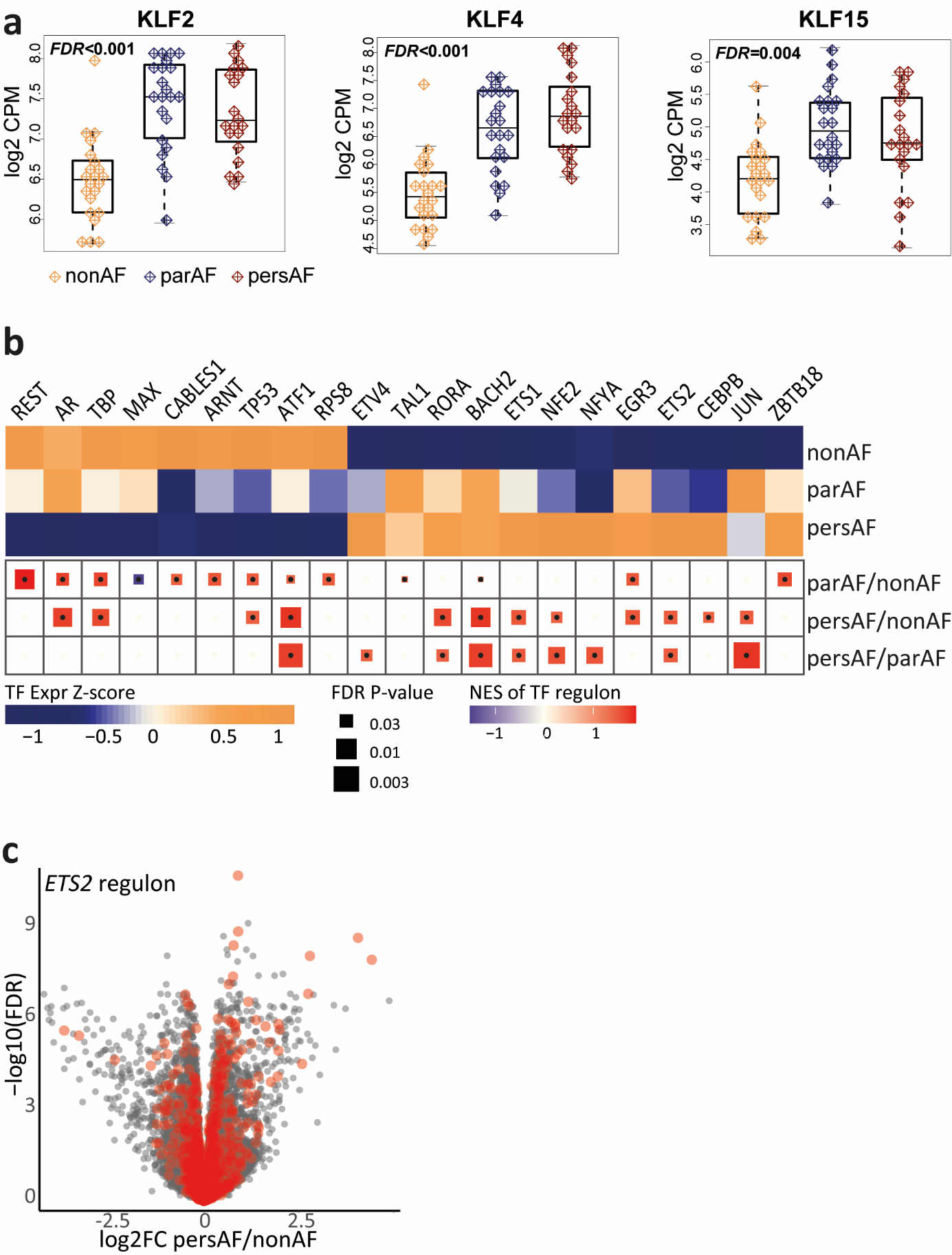
**

**Supplementary Figure S6. ECM gene expression was increased including glycoproteins, proteoglycans, collagens and ECM degradation genes.**

**
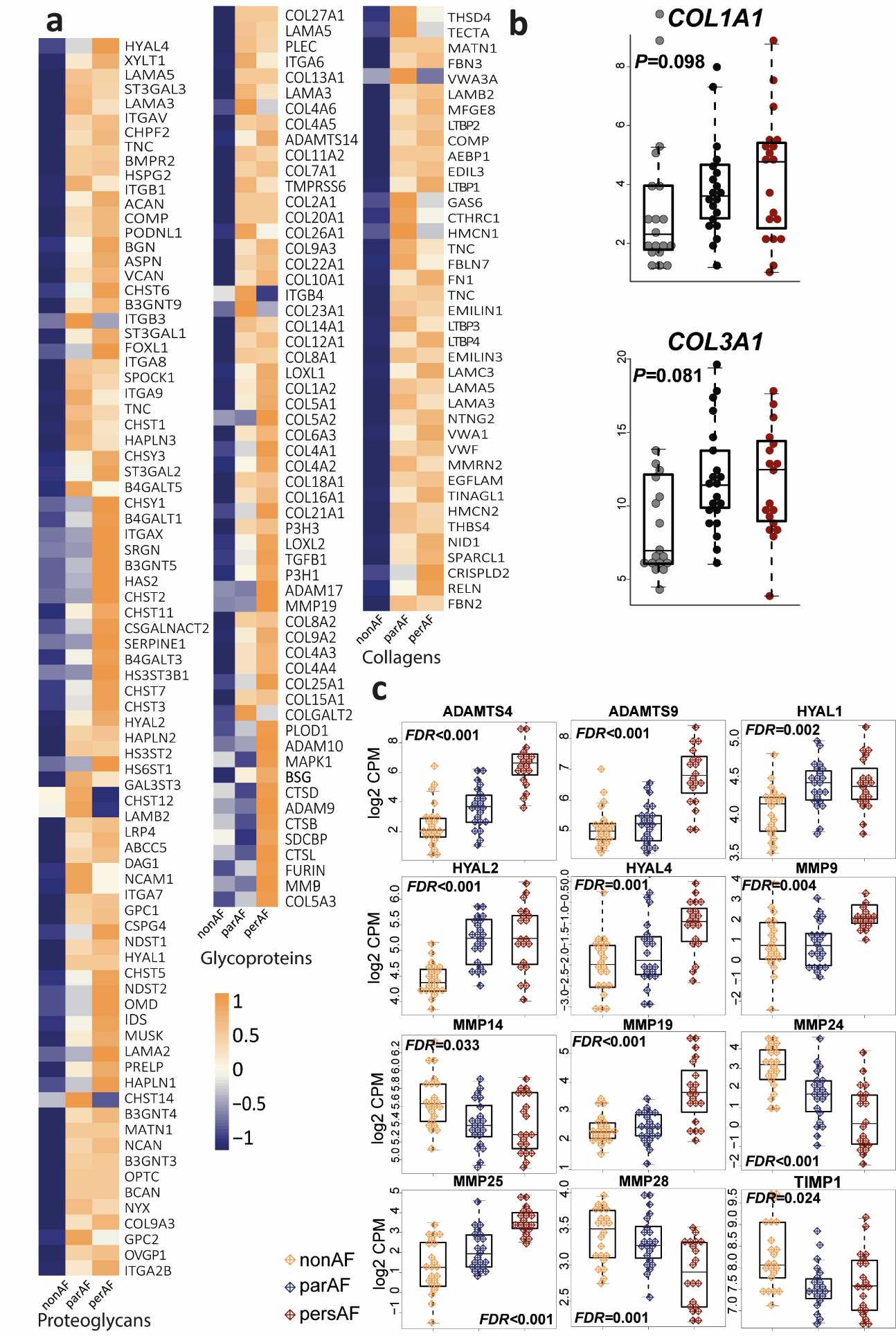
**

**Supplementary Figure S7. KEGG pathway “ECM receptor interaction” demonstrating the regulatory role of cell-matrix interactions and upregulated genes.**


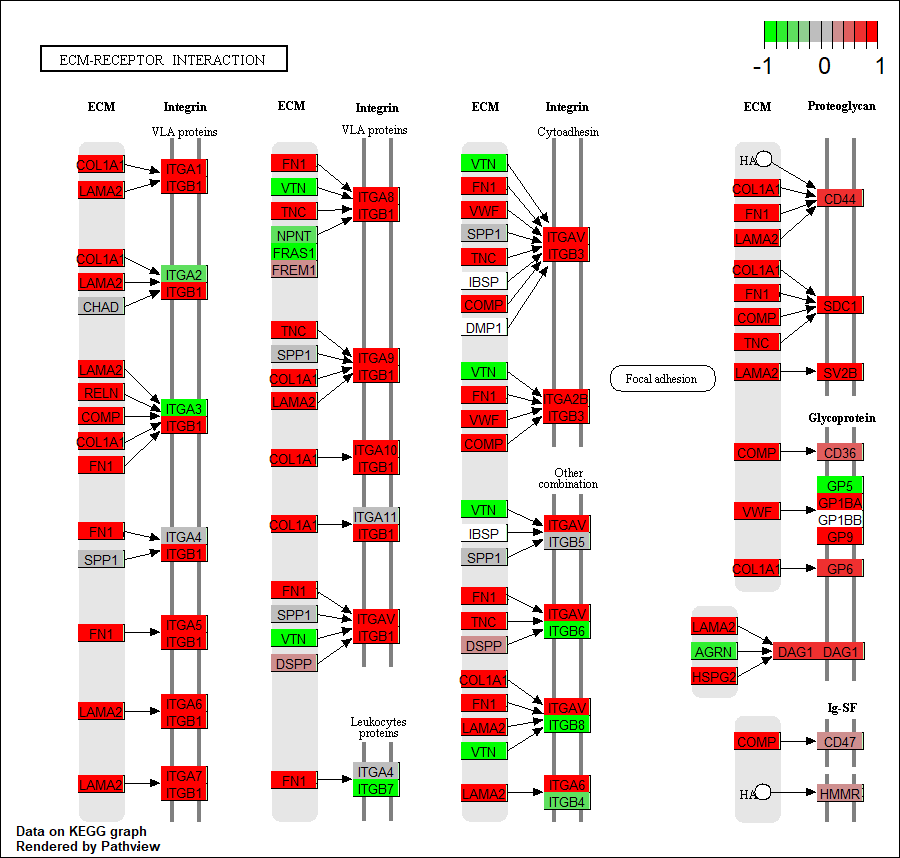


**­­­**

**Supplementary Figure S8. Integrin and focal adhesion gene expression increased in paroxysmal and persistent AF.**

**
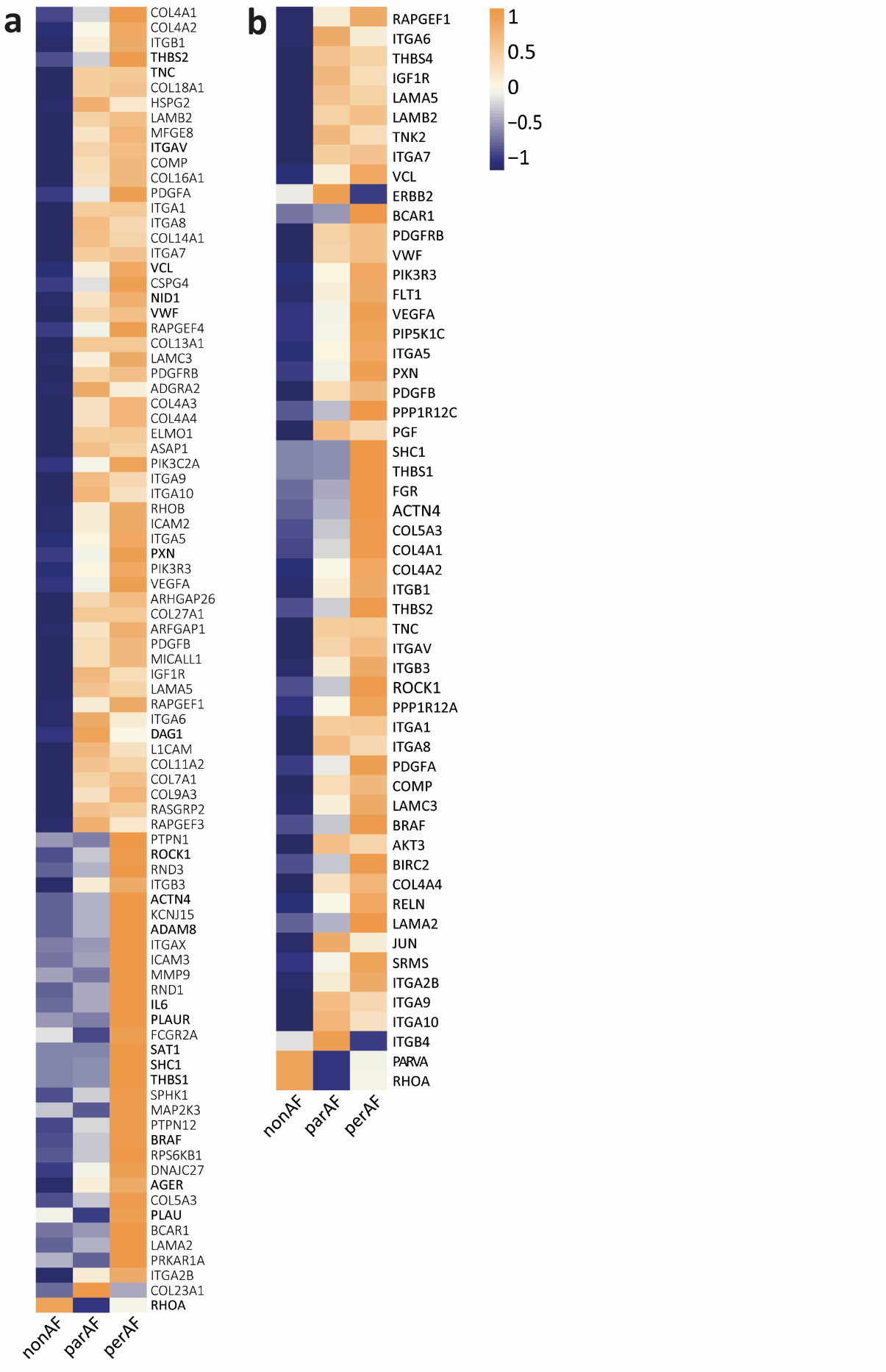
**

**Supplementary Figure S9. KEGG pathway “Focal Adhesion” demonstrating the regulatory role of cell-matrix interactions and upregulated genes.**


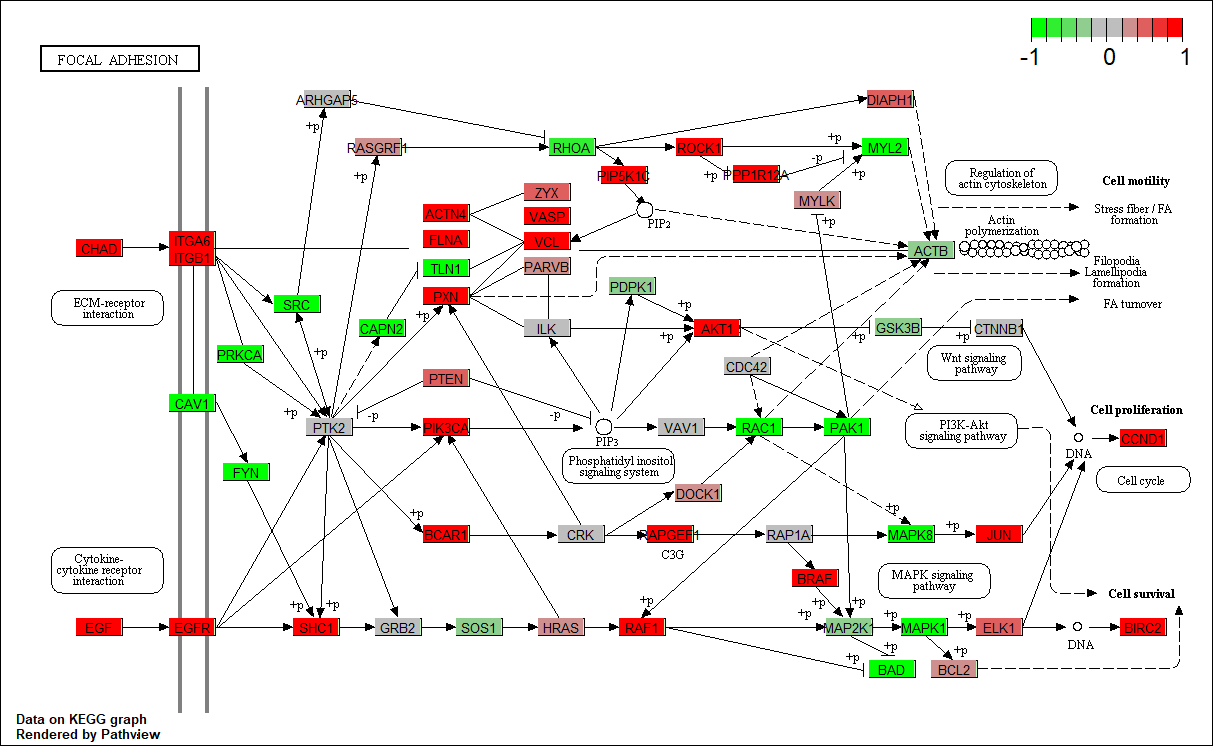


**Supplementary Figure S10. qPCR validated increased PI3K3R expression.**

**
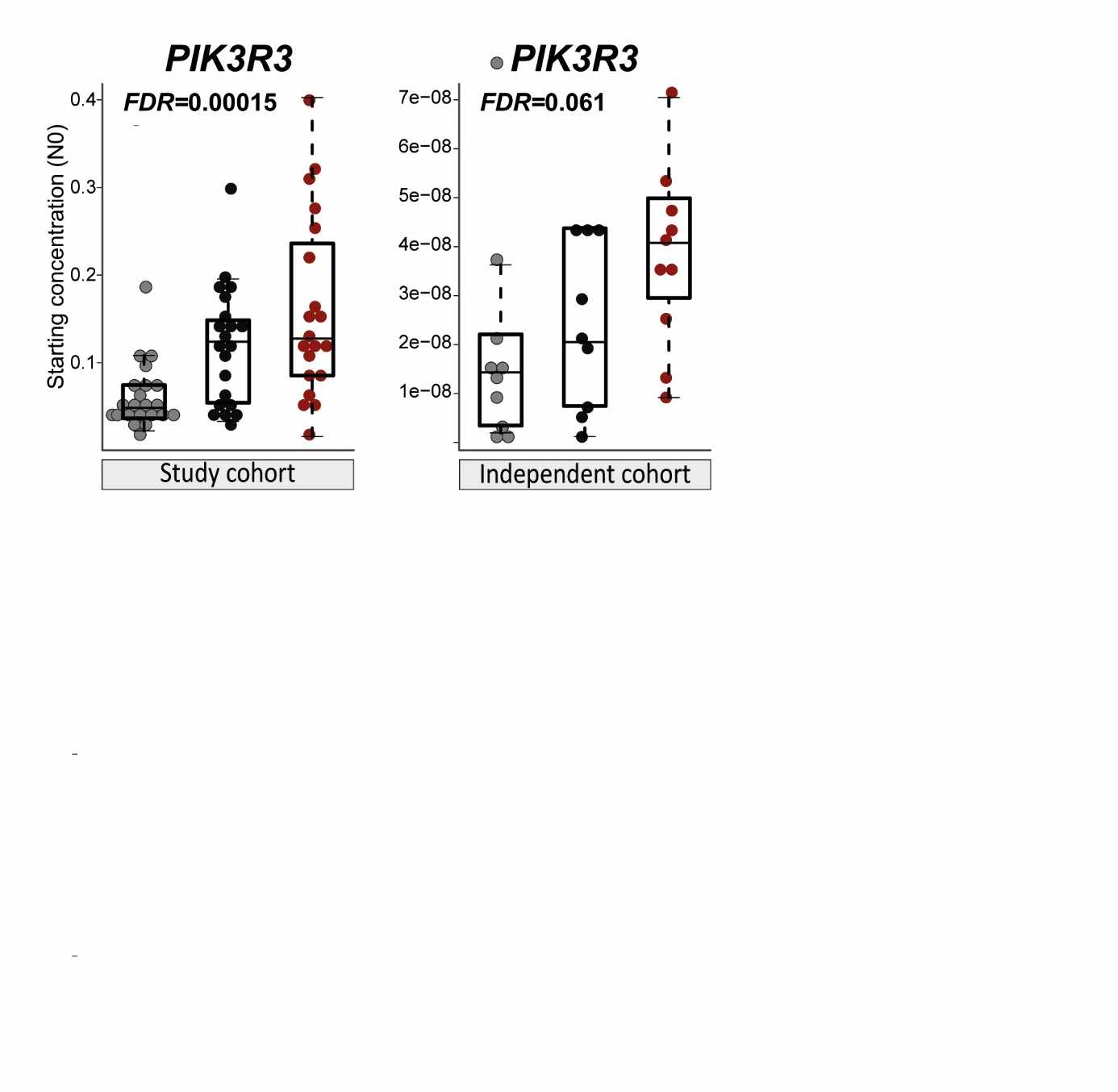
**

**­**

**Figure legends**

**Supplementary Figure S1. Unsupervised hierarchical clustering separates nonAF from persistent AF.** Unsupervised hierarchical clustering of patients almost completely separates non atrial fibrillation patients from persistent atrial fibrillation patients while paroxysmal atrial fibrillation is dispersed in between.

**Supplementary Figure S2. Expression plots of EMT transcription factors.** Various transcription factors have been associated with EMT and were differentially expressed. FDR represent the false discovery rate adjusted *P-*value of the comparison persAF vs. nonAF from the RNA sequencing analysis.

**Supplementary Figure S3. Histological quantification of EAT area reveals no difference between AF and nonAF. a,** overview of left atrial section demonstrating the overall composition of the (sub)epicardium and relation of EAT and fibrosis. Red, EAT; blue, fibrosis covering EAT; green, fibrotic subepicardium not covering EAT. **b,** quantification of EAT area demonstrates no difference between AF and nonAF patients.

**Supplementary Figure S4. NFATC1 was not present in interstitial (endothelial) cells.** Whereas NFATC1 was present in the endocardium and epicardium of persAF patients, no NFATC1+ cells were identified in the interstitium.

**Supplementary Figure S5. Transcription factors associated with angiogenesis. a,** Various Kruppel-like factors were differentially expressed in the sequencing analysis. FDR represent the false discovery rate adjusted *P-*value of the comparison persAF vs. nonAF from the RNA sequencing analysis. **b,** Overview of the TFs that were differentially expressed and demonstrated significant upregulation or downregulation of its regulon by GSEA. Most of these TFs can be associated with angiogenesis. **c,** Volcano plot (persAF vs. nonAF) showing an example of a TF’s transcriptional effects on its regulon. The angiogenesis regulating TF *ETS2* was upregulated along with *ETS2* target genes depicted in red.

**Supplementary Figure S6. ECM gene expression was increased including glycoproteins, proteoglycans, collagens and ECM degradation genes. a,** Heatmaps of leading edge genes involved in the biological processes related to glycoproteins, proteoglycans and collagen biosynthesis. **b,** qPCR validation of *COL1A1* and *COL3A1* demonstrated a tended increase in gene expression in parAF and persAF. **c,** Various genes involved in ECM degradation were differentially expressed. FDR represent the false discovery rate adjusted *P-*value of the comparison persAF vs. nonAF from the RNA sequencing analysis.

**Supplementary Figure S7. KEGG pathway “ECM receptor interaction” demonstrating the regulatory role of cell-matrix interactions and upregulated genes.**

The KEGG pathway “ECM receptor interaction” (hsa04512) was found highly upregulated (perAF/nonAF NES= 2.1; FDR q-Val= 0.0001). The figure demonstrates how and which upregulated ECM components may affect downstream signaling by binding to cell receptors. Scale is normalized based on the ranked gene list used for GSEA.

**Supplementary Figure S8. Integrin and focal adhesion gene expression increased in paroxysmal and persistent AF. a,** Heatmap showing the differentially expressed genes making up the leading edges of all upregulated biological pathways related to integrin signaling. **b,** Heatmap showing the differentially expressed genes of the leading edge of ‘*focal adhesion’* (GO:0005925).

**Supplementary Figure S9. KEGG pathway “Focal adhesion” demonstrating the regulatory role of cell-matrix interactions and upregulated genes.**

The KEGG pathway “Focal adhesion” (hsa04510) was found highly upregulated (perAF/nonAF NES= 1.89; FDR q-Val= 0.019). The figure demonstrates how ECM receptor interactions (Figure S7) have a central regulatory role as they can activate many downstream pathways, which are found upregulated in this study such as *Regulation of actin cytoskeleton* or *PI3K-AKT signaling.* Scale is normalized based on the ranked gene list used for GSEA.

**Figure S10. qPCR validated increased PI3K3R expression.** qPCR expression levels of *PIK3R3.*
